# The Carbapenemase BKC-1 from Klebsiella pneumoniae Is Adapted for Translocation by Both the Tat and Sec Translocons

**DOI:** 10.1128/mBio.01302-21

**Published:** 2021-06-22

**Authors:** Manasa Bharathwaj, Chaille T. Webb, Grishma Vadlamani, Christopher J. Stubenrauch, Tracy Palmer, Trevor Lithgow

**Affiliations:** a Infection & Immunity Program, Biomedicine Discovery Institute and Department of Microbiology, Monash University, Melbourne, Victoria, Australia; b The University of Western Australia, School of Molecular Sciences & The ARC Centre of Excellence in Plant Energy Biology, Crawley, Western Australia, Australia; c Microbes in Health and Disease, Newcastle University Biosciences Institute, Newcastle University, Newcastle upon Tyne, United Kingdom; Institut Pasteur

**Keywords:** β-lactamase, antimicrobial resistance, periplasm, signal peptide, Tat pathway, beta-lactamases, evolution, protein secretion

## Abstract

The cell envelope of Gram-negative bacteria consists of two membranes surrounding the periplasm and peptidoglycan layer. β-Lactam antibiotics target the periplasmic penicillin-binding proteins that synthesize peptidoglycan, resulting in cell death. The primary means by which bacterial species resist the effects of β-lactam drugs is to populate the periplasmic space with β-lactamases. Resistance to β-lactam drugs is spread by lateral transfer of genes encoding β-lactamases from one species of bacteria to another. However, the resistance phenotype depends in turn on these “alien” protein sequences being recognized and exported across the cytoplasmic membrane by either the Sec or Tat protein translocation machinery of the new bacterial host. Here, we examine BKC-1, a carbapenemase from an unknown bacterial source that has been identified in a single clinical isolate of Klebsiella pneumoniae. BKC-1 was shown to be located in the periplasm, and functional in both K. pneumoniae and Escherichia coli. Sequence analysis revealed the presence of an unusual signal peptide with a twin arginine motif and a duplicated hydrophobic region. Biochemical assays showed this signal peptide directs BKC-1 for translocation by both Sec and Tat translocons. This is one of the few descriptions of a periplasmic protein that is functionally translocated by both export pathways in the same organism, and we suggest it represents a snapshot of evolution for a β-lactamase adapting to functionality in a new host.

## INTRODUCTION

The adaptation of a bacterial species to a new niche can be promoted by the acquisition of additional genes by lateral gene transfer (LGT), but this requires that the newly acquired proteins are translated (1), localized (2), and folded into functional forms (3) to deliver the appropriate phenotype (4–7). Antimicrobial resistance (AMR) is such a phenotype and has become of grave concern for human health globally (8, 9). The enzymes that play a key role in the generation of AMR phenotypes in many bacteria are β-lactamases, providing resistance to β-lactam antibiotics. These enzymes are typically active in the periplasm, where they hydrolyze the β-lactam ring of the drug, rendering it inactive (10, 11). Carbapenemases are a subset of β-lactamases effective against the carbapenem class of β-lactam antibiotics used to treat multidrug-resistant bacterial infections (12, 13). In addition to an extended-spectrum β-lactamase (ESBL) resistant phenotype, carbapenemases thereby provide a carbapenem-resistant phenotype, and the widespread use of carbapenems has increased the prevalence of carbapenem-resistant *Enterobacteriaceae* (CRE) (12, 14, 15). There are two prominent types of carbapenemases: (i) the metallo-β-lactamases that require one or more zinc atoms to be coordinated in their active site (e.g., NDM-1), and (ii) the K. pneumoniae carbapenemases that are not metalloenzymes but instead are serine β-lactamases (16). First described in 2001 (17), the most prominent is “Klebsiella pneumoniae carbapenemase 2” or KPC-2 (encoded by *bla*_KPC-2_) and the related variants are the major contributors to widespread CRE phenotypes (14, 18, 19). Genes such as these are found on mobile genetic elements, particularly plasmids, meaning that these genes are readily transmissible (16, 20–23).

To confer an AMR phenotype to the host species, β-lactamases acquired by LGT need to be present at sufficiently high steady-state levels, with this level dependent on the compound efficiency of protein synthesis, protein transport pathways, and protein folding. Thus, the phenotype demands efficiency of host cell processes despite that the “alien” protein from an LGT event did not coevolve with the host cell factors mediating these pathways. For example, there is a global distribution of the CTX-M family of β-lactamases in E. coli (24, 25), K. pneumoniae (26, 27), other *Enterobacteriaceae* (28, 29), and Pseudomonas aeruginosa (30, 31), implying that all of the species acquiring these genes are capable of efficiently translating the polypeptide through what might be nonoptimal codon bias, transporting it across the inner membrane via protein translocases that must recognize its signal peptide, and folding the CTX-M protein so that the functional form populates the periplasm. Studies directed at the CTX-M β-lactamases show that this requires the translocation of the nascent polypeptide across the inner membrane in an unfolded state, followed by protein folding reactions in the periplasm to generate the enzymatically active β-lactamase (32–34).

Most of the proteins that function in the periplasm are translocated across the inner membrane by the general secretory (Sec) pathway as linear polypeptide chains, and their folding in the periplasm is facilitated by a specific set of chaperones (35–38). In order to engage the Sec translocon, protein substrates possess an N-terminal signal peptide of about 17 to 24 amino acids in length, comprising a positively charged n-region, a highly hydrophobic h-region, and a polar c-region that includes the cleavage site for signal peptidase (39, 40). Given that the first serine β-lactamases evolved from penicillin binding proteins (41, 42), which utilize the Sec pathway for their translocation to the periplasm (43), it was assumed that these enzymes would also translocate via Sec. This assumption proved to be valid for most β-lactamases, including TEM-1, CTX-M-14, and AmpC, each of which have been widely distributed among drug-resistant *Enterobactericeae* (29, 44–47).

Given this paradigm, it was curious to see that a small cohort of chromosomally encoded serine β-lactamases, such as L2 from Stenotrophomonas maltophilia and BlaC from Mycobacterium tuberculosis, are not transported by the Sec translocon, but instead utilize the twin arginine translocase (Tat) machinery for translocation across the inner membrane (47, 48). This means that these few β-lactamases use an alternate pathway where they are first folded in the cytoplasm and only thereafter translocated as fully functional enzymes (47).

Substrate proteins that engage the Tat machinery possess an N-terminal signal peptide that is usually between 27 and 35 amino acids in length and that contains both Tat-targeting and Sec-avoidance features to ensure the exclusivity that is known to exist for these protein translocation pathways (49, 50). The major Tat recognition feature is a pair of almost invariant arginine residues in the signal peptide n-region, found as part of the SRRXFL motif (49, 51). A combination of an h-region that is only moderately hydrophobic, coupled with the presence of one or more positively charged residues in either the c-region or at the N terminus of the mature protein, serve as Sec avoidance elements (52–55). Thus, signal peptides have evolved to target their passenger proteins strictly to either the Tat or Sec machinery. For protein substrates acquired through LGT, this also matters in terms of the folding assistance they will be provided in the new host cell; intrinsic sequence and structural features that require folding by the chaperones in the periplasm posttranslocation (35, 36, 39) will only be satisfied if the protein is translocated in an unfolded state by the Sec translocon. Conversely, intrinsic sequence and structural features that require folding in the cytoplasm prior to translocation will only be satisfied if the protein is translocated in a folded state by the Tat translocon (56, 57). While recent studies suggest there is some leeway for overlapping features that define Sec or Tat signal peptides (53, 55), with the exception of the Sec-dependent lipase in Bacillus subtilis (58), which can overflow into the Tat pathway upon multicopy overproduction, there is very limited evidence that a single protein is capable of using both translocons in the same organism.

Initial studies on the signal peptides of β-lactamases revealed three putative Tat-transported enzymes: BKC-1 and GPC-1, first identified in clinical isolates of K. pneumoniae and P. aeruginosa, respectively (59–61), as well as PAD-1 from the soil bacterium Paramesorhizobium desertii (60). Here, we show that these three proteins represent an isolated subfamily of β-lactamases with carbapenemase activity. Furthermore, through the production of BKC-1 in a model strain of E. coli with or without a functional Tat system, we show that while a subpopulation of protein molecules are translocated by the Sec pathway, the rest are translocated via Tat translocon. Therefore, mounting a carbapenem-resistant phenotype depends on the presence of the Tat machinery to provide sufficient translocation of BKC-1 into the periplasm.

## RESULTS

### Equivalent plasmids carry *bla*_BKC-1_ and *bla*_KPC-2_.

**FIG 1:**
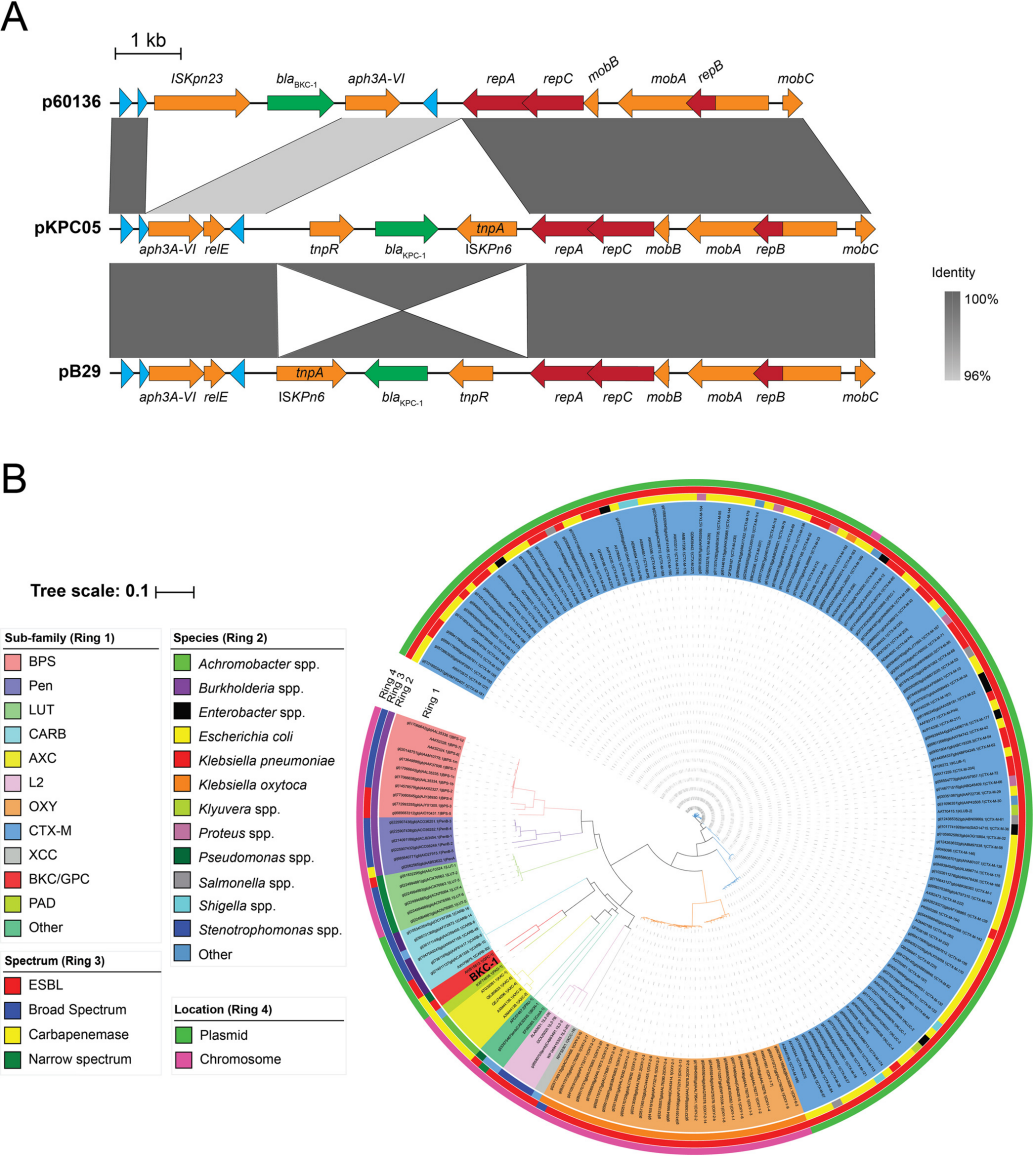
Comparison of the genetic environment of BKC-1. (A) The *bla*_BKC-1_ gene is carried on an RSF1010 plasmid, p60136 (59), the sequence of which was aligned with two related IncQ plasmids from K. pneumoniae (pKPC05 with 87.58% overall sequence identity and pB29 with 84.61% overall sequence identity) using Easyfig 2.2.5. Arrows represent genes as follows: β-lactamase encoding genes (green); replication-related genes (red); other genes of known function (orange); and genes of unknown function (blue). (B) A global phylogeny of class A β-lactamases was constructed by sequence comparison of class A β-lactamases from BLDB. The sequences displayed were retrieved from BLDB allowing for at least 40% sequence identity. The position of BKC-1 is denoted in bold. GPC-1, a β-lactamase identified in P. aeruginosa, showed the greatest amino acid sequence identity to BKC-1 (77%), while the next closest relationship (63% sequence identity) was with the chromosomally encoded β-lactamase (PAD-1) from P. desertii. The four rings that designate features in the phylogeny are as indicated in the legend.

The plasmid p60136 harboring *bla*_BKC-1_ was identified in a carbapenem-resistant Klebsiella isolate (59). Gene signatures, including the transposable element IS*Kpn23* (Fig. 1A), were suggestive of a gene transposition from another species via LGT. Sequence analyses revealed that p60136 is related to the globally disseminated pB29 (GenBank accession MK330869) and pKPC05 (GenBank accession MK330868) plasmids. However, rather than *bla*_BKC-1_, these other two IncQ plasmids carry *bla*_KPC-2_ flanked by elements from the transposon Tn*4401*, the transposase (*tnpA*) from IS*Kpn6* and the *tnpR*-encoded resolvase. Apart from these transposon elements that are characteristic of *bla*_KPC-2_-harboring plasmids (62), the three plasmids have a high degree of sequence identity, including across the replication genes (Fig. 1A).

We sought to understand the overall sequence relationships between BKC-1 and other β-lactamases that have been identified globally. Using a 40% sequence identity cutoff as an indicator of diverse β-lactamases, sequences were extracted from BLDB (Beta-lactamase database) (63) and the top 200 hits were subsequently analyzed using iTOL (64) (Fig. 1B). Molecular phylogeny analysis in this global context demonstrated that BKC-1 is most closely related to GPC-1 from P. aeruginosa (61), and these two in turn are related to PAD-1 from the soil bacterium P. desertii (60). These proteins have a sequence identity of 66% (Fig. 1B), but no other closely related proteins are present in the BLDB.

### Targeting and subcellular localization of BKC-1.

**FIG 2:**
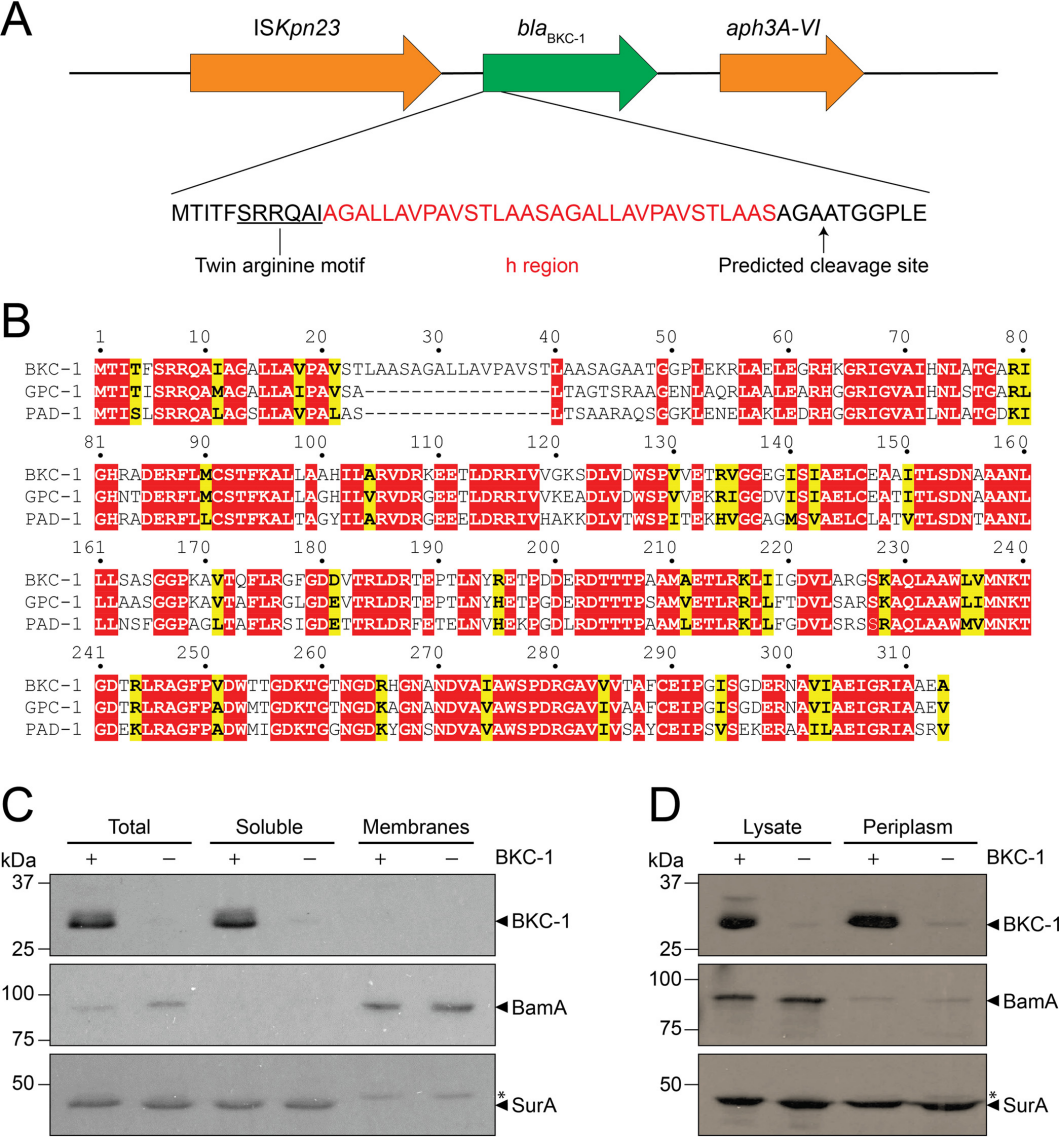
A unique signal sequence targets BKC-1 into the periplasm. (A) The position and sequence of the signal peptide encoded in *bla*_BKC-1_. The twin arginine motif (underlined), 32-residue h-region (red), and cleavage site (AGA-AT) are indicated. (B) Protein sequence alignment of BKC-1 from K. pneumoniae, GPC-1 from P. aeruginosa, and PAD-1 from P. desertii. Red indicates identical residues; yellow indicates conserved residues. (C and D) Cell lysates from E. coli transformed with pJPCmR (–) or pJPBKC-1His encoding C-terminally His_6_-tagged BKC-1 (+) were fractionated (C) or TSE-extracted (D) and analyzed by SDS-PAGE and immunoblotting using antibodies raised against BKC-1, BamA (membrane protein control), or SurA (periplasmic protein control). Asterisks indicate the slower-migrating nonspecific protein present in SurA immunoblots.

The vast majority of bacterial signal peptides are 20 to 30 amino acids long (65). However, initial sequence analysis revealed an unexpected feature in the N-terminal region of nascent BKC-1, where an unusually long, 46-residue signal peptide that included a twin arginine motif (SRRQAI) and a putative cleavage site after Ala46 was present (Fig. 2A). A multiple sequence alignment of BKC-1 from K. pneumoniae with GPC-1 from P. aeruginosa and PAD-1 from P. desertii showed that a 16-residue duplication in the h-region of the signal peptide of BKC-1 is responsible for its increased length (Fig. 2B; Fig. S1 in the supplemental material).

10.1128/mBio.01302-21.2 FIG S1 Alignment of *bla*_BKC-1_ with closely related genes. Nucleotide sequence alignment of *bla*_BKC-1_ from K. pneumoniae, *bla*_GPC-1_ from P. aeruginosa, and *bla*_PAD-1_ from *P. desertii*. Red highlighting represents conserved nucleotides. Download FIG S1, PDF file, 1.1 MB. Copyright © 2021 Bharathwaj et al. 2021 Bharathwaj et al. https://creativecommons.org/licenses/by/4.0/ This content is distributed under the terms of the Creative Commons Attribution 4.0 International license.

To investigate the biochemical impact of these signal sequence features in BKC-1, we used the well-characterized E. coli model system to study its targeting and translocation. To determine whether BKC-1 activity in E. coli was similar to that seen in K. pneumoniae, the coding sequence of *bla*_BKC-1_ was cloned under either a constitutive TetR promoter in pACYC184 (66) or a tetracycline-inducible promoter in pJPCmR (67). K. pneumoniae B5055 and E. coli BW25113 were transformed with each plasmid and antibiotic sensitivity was assessed by MIC assays. In addition to ampicillin, the broad-spectrum cephalosporin drugs ceftriaxone, ceftazidime, and cefotaxime were tested, as were the carbapenems imipenem, meropenem, and ertapenem. All BKC-1-expressing strains had similar levels of resistance toward each β-lactam tested (Table 1). The MIC values showed similar trends for both species regardless of whether the carbapenemase was under the control of a constitutive or inducible promoter. MICs for cells expressing BKC-1 were either at or below the breakpoint for resistance for all three carbapenems and ceftazidime (Table 1), while MICs for ampicillin, ceftriaxone, and cefotaxime were higher than the breakpoint, consistent with previous findings that BKC-1 does not hydrolyze carbapenems and ceftazidime as efficiently as other antibiotics tested (59). Growth curve analysis of E. coli BW25113 harboring plasmid-encoded BKC-1 showed that its expression did not impose a fitness cost (Fig. S2), whereas for K. pneumoniae B5055, a moderately lower growth rate was observed (Fig. S2).

**TABLE 1:** MIC assessment of E. coli BW25113 and K. pneumoniae B5055 expressing BKC-1

Drug	MIC (μg/ml)										
	E. coli BW25113					K. pneumoniae B5055					
	No plasmid	Constitutive		Induced		No plasmid	Constitutive		Induced		Break point^*a*^
		pACYC184	+BKC-1	pJPCmR	+BKC-1		pACYC184	+BKC-1	pJPCmR	+BKC-1	
Ampicillin	4	4	>1,024	4	>1,024	4	4	>1,024	4	>1,024	≥32
Ceftriaxone	≤0.0625	≤0.0625	64	≤0.0625	>64	≤0.0625	≤0.0625	64	≤0.0625	>64	≥4
Cefotaxime	≤0.0625	≤0.0625	64	≤0.0625	>64	≤0.0625	≤0.0625	32	≤0.0625	64	≥4
Ceftazidime	0.25	<0.25	4	0.25	8	0.25	0.25	8	0.25	8	≥16
Imipenem	0.125	0.125	2	0.125	4	0.5	0.5	4	0.5	4	≥4
Meropenem	0.03125	0.03125	1	0.03125	1	0.03125	0.03125	1	0.03125	1	≥4
Ertapenem	≤0.015	≤0.015	0.5	≤0.015	0.5	≤0.015	≤0.015	1	≤0.015	1	≥2

a CLSI M100-ED30 breakpoint.

10.1128/mBio.01302-21.3 FIG S2 Growth curve analysis of E. coli and K. pneumoniae expressing *bla*_BKC-1_. (A and B) Growth curves of E. coli BW25113 with inducible *bla*_BKC-1_ constructs in the absence (A) and presence (B) of the inducer ATc. (C and D) Growth curves of K. pneumoniae B5055 with inducible *bla*_BKC-1_ constructs in the absence (C) and presence (D) of the inducer ATc. (E and F) Growth curves of E. coli BW25113 (E) and K. pneumoniae B5055 (F) with constitutively expressing *bla*_BKC-1_ constructs. (*n* = 3, error bars represent standard deviations). Download FIG S2, PDF file, 0.7 MB. Copyright © 2021 Bharathwaj et al. 2021 Bharathwaj et al. https://creativecommons.org/licenses/by/4.0/ This content is distributed under the terms of the Creative Commons Attribution 4.0 International license.

To determine the localization of BKC-1 in E. coli BW25113, subcellular fractionation was undertaken using lysates prepared from cells expressing C-terminally hexahistidine (His_6_)-tagged BKC-1 following 4 h of anhydrotetracycline (ATc) induction. Immunoblotting confirmed BKC-1 to be present exclusively in the soluble cellular fraction (combined cytoplasm and periplasm), and absent from membranes (Fig. 2C). This finding indicates that the unusually long signal sequence of BKC-1 is not acting as an inner membrane anchor. To confirm that the protein was periplasmic, osmotic shock was used to release the periplasmic contents and immunoblotting (Fig. 2D) confirmed BKC-1 to be localized to the periplasm.

### Loss of TatC diminishes β-lactam resistance conferred by BKC-1.

TatC is an essential component of the Tat system that interacts with substrate proteins via the twin arginine motif of the signal peptide (68). This binding step initiates the full assembly of the Tat translocon and subsequent transport of substrate across the inner membrane (50, 69). Sequence analysis suggested the signal peptide of BKC-1 has features consistent with the protein being a substrate of the Tat pathway (Table 2). To assess the role of the Tat system in β-lactamase translocation into the periplasm, C-terminally His_6_-tagged BKC-1 was expressed in BW25113 and/or the isogenic Δ*tatC* mutant (70). As controls, the β-lactamases KPC-2 (putatively Sec dependent) and L2 (Tat dependent) were concomitantly expressed with a His_6_ epitope incorporated at the C terminus of each protein. The expression of these β-lactamases did not compromise the growth of E. coli BW25113 nor its isogenic Δ*tatC* mutant (Fig. S3), except that L2 imposed minor fitness defects.

**TABLE 2:** Signal sequence analysis of BKC-1, BKC-1A, KPC-2, and L2

Protein	Signal sequence^*b*^	Tat probability score	Maximum S score^*a*^
BKC-1	MTITF**SRRQAI**AGALLAVPAVSTLAASAGALLAVPAVSTLAASAGA	0.84	0.843
BKC-1A	MTITF**SRRQAI**AGALLAVPAVSTLAASAGA	0.93	0.853
KPC-2	MSLYRRLVLLSCLSWPLAGFSATA	0.01	0.223
L2	MLA**RRRFLQ**FSGAAVASSLALPLLARAAGKATANA	0.99	0.841

a Score obtained from TatP 1.0 (cutoff 0.75).

b Twin arginine motifs are in boldface type and h regions are underlined.

10.1128/mBio.01302-21.4 FIG S3 Growth curve analysis of E. coli expressing various β-lactamases. Growth curves of E. coli BW25113 (A and B) or its isogenic Δ*tatC* mutant (C and D) with inducible *bla* constructs in the absence (A and C) and presence (B and D) of the inducer ATc. The indicated β-lactamases contain a C-terminal His_6_ tag. (*n* = 3, error bars represent standard deviations). Download FIG S3, PDF file, 0.7 MB. Copyright © 2021 Bharathwaj et al. 2021 Bharathwaj et al. https://creativecommons.org/licenses/by/4.0/ This content is distributed under the terms of the Creative Commons Attribution 4.0 International license.

As a clinically relevant readout for enzymatically active β-lactamases, antibiotic sensitivity to ampicillin, three cephalosporins, and three carbapenems was assessed using MIC assays in the presence of anhydrotetracycline (ATc) (Table 3). Surprisingly, the phenotype of the Δ*tatC* mutant producing BKC-1 was drug dependent. In the case of ceftazidime, detectable resistance conferred by BKC-1 was strictly dependent on the presence of TatC (Table 3). However, resistance to cefotaxime, ampicillin, and ceftriaxone, although reduced in the Δ*tatC* mutant, was not completely abolished by inactivation of the Tat pathway (Table 3). MICs of Δ*tatC* mutant cells expressing BKC-1 were reduced by more than 2-fold for ampicillin, ceftriaxone, and cefotaxime, 32-fold for ceftazidime, and 2-fold for all three carbapenems tested. These observations suggest that the transport of BKC-1 is, at least in part, dependent on the Tat system; however, there is clearly some translocation of the enzyme in a Tat-independent manner.

**TABLE 3:** MIC assessment of E. coli expressing BKC-1, KPC-2, and L2 in the presence and absence of TatC

Drug	MIC (μg/ml)									
	BW25113					BW25113 Δ*tatC*				
	No plasmid	Induced				No plasmid	Induced			
		pJPCmR	+BKC-1	+KPC-2	+L2		pJPCmR	+BKC-1	+KPC-2	+L2
Ampicillin	4	4	>1,024	>1,024	>1,024	4	4	512	>1,024	4
Ceftriaxone	≤0.0625	≤0.0625	>64	32	>64	≤0.0625	≤0.0625	64	32	≤0.0625
Cefotaxime	≤0.0625	≤0.0625	>64	16	64	≤0.0625	≤0.0625	32	16	≤0.0625
Ceftazidime	0.25	0.25	8	8	32	0.25	0.25	0.25	8	0.25
Imipenem	0.125	0.125	4	8	NT^*a*^	0.125	0.125	2	8	NT
Meropenem	0.03125	0.03125	1	4	NT	≤0.015	≤0.015	0.5	4	NT
Ertapenem	≤0.015	≤0.015	0.5	4	NT	≤0.015	≤0.015	0.25	4	NT

a NT, not tested, because L2 is not a carbapenemase.

The substrate-activity profile for BKC-1 revealed that the enzyme has marginal activity toward ceftazidime relative to the other β-lactam antibiotics tested (MIC of 8 μg/ml compared to at least 64 μg/ml for the other drugs shown in Table 3). This suggests that the decreased level of BKC-1 accumulating in the periplasm in the absence of TatC is likely sufficient to hydrolyze β-lactam drugs that are good substrates for the enzyme, but insufficient to turn over drugs such as ceftazidime, for which the enzyme is less well suited. As expected, KPC-2 activity as revealed through MIC values was unaffected in the Δ*tatC* mutant strain, confirming its translocation to be independent of the Tat system. In contrast, L2 activity was strictly dependent on TatC and the sensitivity of the Δ*tatC* mutants was 64-fold less, for all four drugs tested. This is consistent with previously findings that L2 is exclusively a substrate of the Tat translocon and its translocation does not rely on the Sec pathway (47).

**FIG 3:**
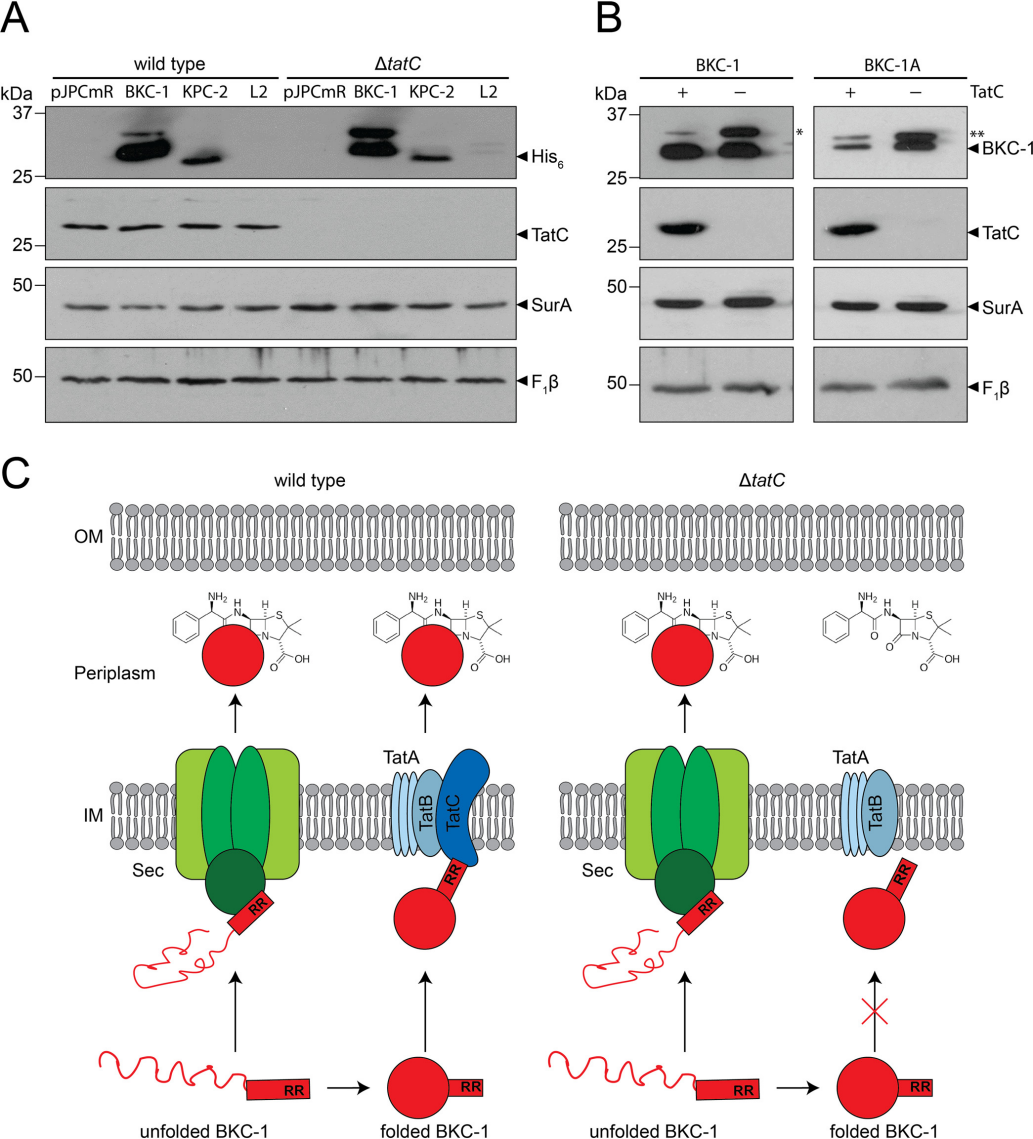
The unique signal sequence of BKC-1 is required for efficient Tat-independent translocation into the periplasm. (A and B) Whole-cell lysates were prepared from wild-type E. coli BW25113 or its isogenic Δ*tatC* mutant to compare levels of BKC-1 with other β-lactamases (A) or its BKC-1A derivative that has a shorter signal peptide (B). pJPCmR or its derivative vectors containing C-terminally His_6_-tagged β-lactamases proteins were used to synthesize the indicated protein of interest (above immunoblot). The extracts were analyzed by SDS-PAGE and immunoblotting using antibodies raised against the indicated proteins (right of immunoblot). SurA and F_1_β serve as loading controls. Asterisks indicate precursor protein forms that migrate slower by SDS-PAGE. (C) The proposed model for the function and translocation of BKC-1 across the inner membrane (IM) in the presence and absence of TatC. The topological compartments of the periplasm and outer membrane (OM) are indicated. The unfolded and folded forms of BKC-1 (red) are shown and the rectangle with twin arginines (RR) represents its signal peptide. In the periplasm, both translocated forms of BKC-1 work together to hydrolyze β-lactam antibiotics, but in the absence of TatC, there is a decreased number of BKC-1 that can enter the periplasm.

To support these observations, immunoblotting was undertaken on whole-cell samples. Even following overexposure, the His_6_-tagged epitope on L2 could not be detected in either wild-type or Δ*tatC* mutant backgrounds (Fig. 3A). This suggests that the tag may be proteolytically removed from this construct, since the protein is clearly present and functional, as judged by the phenotypes displayed in the MIC evaluation of these strains (Table 3). The level of KPC-2 expression appeared to be relatively similar in wild-type and Δ*tatC* mutant cells (Fig. 3A), also consistent with the phenotype observed from MIC experiments. In the *tatC*^+^ background, BKC-1 appeared to be present at higher levels than KPC-2, and the vast majority of the protein was present as the processed, mature form (Fig. 3A). In contrast, in the Δ*tatC* mutant, although some tagged BKC-1 of the mature size was detected, a slower-migrating form of the protein accumulated, at the expected size of the cytoplasmic unprocessed precursor. This is consistent with reduced translocation into the periplasm in the absence of a functional Tat pathway and is consistent with the reduced resistance phenotypes for ampicillin, ceftriaxone, and cefotaxime, as well as the Tat dependence seen for ceftazidime resistance (Table 3).

### Signal peptide modification of BKC-1.

The results presented above suggested that the targeting of BKC-1 is more complex than observed for other β-lactamases, being partially dependent on both Sec and Tat pathways. To address the extent to which the function of BKC-1 depends on its long signal sequence, we used GPC-1 and PAD-1 as models (Fig. 2B) to construct a modified sequence of BKC-1 (termed BKC-1A), where the 16-residue duplication of the h-region was removed. Expression of BKC-1A in E. coli BW25113 and its isogenic Δ*tatC* mutant imposed very minor fitness defects, similar to L2 expression (Fig. S3), but cell densities and growth rates were comparable to wild-type cells. We next assessed the phenotypic impact of truncating the BKC-1 signal peptide by measuring MICs using the same seven antibiotics as before in both the wild-type and the Δ*tatC* mutant background (Table 4). Although the 16-residue deletion did not affect resistance to ampicillin, the deletion did alter the resistances to all other drugs tested by at least 2-fold. MICs for cephalosporins with wild-type cells expressing BKC-1A were 2- to 4-fold lower than those expressing the native BKC-1 (Table 4). These effects were even more pronounced in the absence of TatC, where the MICs were 2-fold, 4-fold, and 8-fold lower for ceftriaxone, ceftazidime, and cefotaxime, respectively, compared to wild-type cells expressing BKC-1A. Carbapenem MICs were also 2-fold lower for cells expressing BKC-1A compared to those that expressed BKC-1 (Table 4), and this difference was preserved in the absence of TatC. Overall, this suggests that BKC-1 might be better suited for delivering a cephalosporin resistance phenotype than BKC-1A in the presence or absence of TatC.

**TABLE 4:** MIC assessment of E. coli expressing BKC-1 and BKC-1A in the presence and absence of TatC

Drug	MIC (μg/ml)							
	BW25113				BW25113 Δ*tatC*			
	No plasmid	Induced			No plasmid	Induced		
		pJPCmR	+BKC-1	+BKC-1A		pJPCmR	+BKC-1	+BKC-1A
Ampicillin	4	4	>1,024	>1,024	4	4	512	512
Ceftriaxone	≤0.0625	≤0.0625	>64	64	≤0.0625	≤0.0625	64	32
Cefotaxime	≤0.0625	≤0.0625	>64	64	≤0.0625	≤0.0625	32	16
Ceftazidime	0.25	0.25	8	2	0.25	0.25	0.25	0.25
Imipenem	0.125	0.125	4	2	0.125	0.125	2	1
Meropenem	0.03125	0.03125	1	0.5	≤0.015	≤0.015	0.5	0.25
Ertapenem	≤0.015	≤0.015	0.5	0.25	≤0.015	≤0.015	0.25	0.125

To assess the relative levels of the carbapenemase in cells producing either BKC-1 or BKC-1A, immunoblotting was performed with whole-cell lysates using an antibody raised against BKC-1 (Fig. 3B). In each case, it appears there is more carbapenemase present in cells lacking TatC than in wild-type cells. For BKC-1, this increase in protein level does not appear to drastically affect the mature (periplasmic localized) form of the protein, but corresponds to an abundance of the precursor form of BKC-1. However, for BKC-1A, levels of precursor and mature forms of the protein are comparable, but there is a marked reduction in the amount of mature protein in the absence of a functional Tat translocon. Additionally, BKC-1A levels appeared to be lower than those of BKC-1 in the presence or absence of TatC (Fig. 3B). This is consistent with the MIC data (Table 4), where the expression of BKC-1 favors a more resistant phenotype than BKC-1A.

### Assessing the importance of the twin arginine motif in BKC-1 in conferring resistance to β-lactams in K. pneumoniae.

In order to determine whether the twin arginine motif was required for BKC-1 to confer a β-lactam resistant phenotype in K. pneumoniae, the organism in which BKC-1 was discovered (59), we modified the signal peptide of BKC-1 by substituting the essential arginines for two lysines, a substitution known to abolish interaction with the Tat translocon (55, 69). Because we noted that BKC-1-dependent ceftazidime resistance in E. coli was dependent on a functional Tat translocon (Table 3 and 4), we assessed whether expression of this modified variant, BKC-1KK, in K. pneumoniae B5055 would similarly fail to confer resistance to ceftazidime. Indeed, the MIC measured in cells producing this variant was indistinguishable from cells lacking BKC-1 (MIC of 0.25 μg/ml; Table 5). While this signal peptide substitution prevents Tat translocation, it did not prevent BKC-1KK from being targeted to the Sec translocon because ampicillin resistance was not affected (MIC of >1,024 μg/ml; Table 5). We then assessed whether the 16-residue deletion in the signal peptide h-region (BKC-1A) would alter ceftazidime MICs in K. pneumoniae, and again found a 4-fold reduction (i.e., MIC of 2 μg/ml) compared to native BKC-1, similar to what was observed for E. coli (Table 4). Overall, this demonstrates that ceftazidime resistance conferred by BKC-1 is dependent on the Tat translocon in both K. pneumoniae and E. coli.

**TABLE 5:** MIC assessment of K. pneumoniae B5055 expressing BKC-1, BKC-1A and BKC-1KK

Drug	MIC (μg/ml)				
	No plasmid	Induced			
		pJPCmR	+BKC-1	+BKC-1A	+BKC-1KK
Ampicillin	4	4	>1,024	>1,024	>1,024
Ceftriaxone	≤0.0625	≤0.0625	>64	>64	>64
Cefotaxime	≤0.0625	≤0.0625	64	64	32
Ceftazidime	0.25	0.25	8	2	0.25
Imipenem	0.5	0.5	4	2	4
Meropenem	0.03125	0.03125	1	1	1
Ertapenem	≤0.015	≤0.015	1	0.5	0.5

## DISCUSSION

Theoretically, there are a number of restrictions to the dissemination of AMR and virulence phenotypes in bacteria by LGT (4, 5, 7). Our initial interest in BKC-1 was piqued by the observation that two related enzymes, BKC-1and GPC-1, have each been identified only once and, despite being carried by highly transmissible plasmids, have not spread further by LGT or arisen more broadly than in the distinct countries where each was first found (59–61, 71). With only a few related sequences available, it is difficult to trace the exact origins of this lineage of carbapenemases, but a very recent report suggested that the plasmid-encoded BKC-1 may have originated from *Shinella*, given the oddity of its sequence (72). Whatever the ancestral reservoir for BKC-1 and related carbapenemases, genes transferred by LGT are often initially silenced by regulatory factors since they pose a potential risk to the host bacterium (1, 7, 73). It has been argued previously that one such risk to the host is that codon usage differences would impact on translation rates in these naive host species, or that protein translocation rates in cells expressing proteins acquired through LGT will block the biosynthetic machinery (74). Indeed, studies in E. coli showed that the overall assembly rates for virulence factors like FimD and UshC are dependent on how well features in these protein sequences can be handled by the components of the host cell’s biosynthetic machinery (74). Similarly, for β-lactamases, a substantial fitness cost of carbapenem resistance has been quantified and linked to amino acid starvation due to codon usage incompatibility and other issues associated with maintaining sufficiently high expression levels of the carbapenemase (75).

For enzymes such as β-lactamases that function in the periplasm, translocation across the inner membrane can be achieved via either of two protein complexes, Sec or Tat (39, 50). Here, the carbapenemase KPC-2 was found to engage with the Sec system for translocation into the periplasm, where it is presumably folded by periplasmic chaperones such as DsbA, which helps introduce disulfide bridges that are known to stabilize carbapenemases for optimum carbapenem hydrolysis (76), as is the paradigm for β-lactamase assembly reported in *Enterobacteriaceae* (47). Conversely, the Tat machinery translocates proteins folded in the cytoplasm, and L2 activity in the periplasm was shown to be wholly dependent on a functional Tat system, as reported previously (47). The increased sensitivity to β-lactams of E. coli harboring BKC-1 in the absence of TatC indicates that BKC-1 uses both translocation systems. Based on our findings, we suggest a dual-translocon targeting model for the export of BKC-1 (Fig. 3C). An interpretation of our data in terms of this model would be that BKC-1 is not yet especially adapted to either Sec or Tat in *Enterobacteriaceae*, but can deliver a highly β-lactam resistant phenotype only by using both arms of the host cell’s protein translocation machinery. This implies that a subset of precursor BKC-1 remains unfolded in the cytoplasm (likely due to interactions with cytoplasmic chaperones), translocates via the Sec translocon, and later folds in the periplasm to assume its functional form, while the remaining subset of precursor BKC-1 folds in the cytoplasm, translocates via the Tat translocon, and assumes its functional form in the periplasm.

Experiments with BKC-1 and the truncated BKC-1A suggest that the extended signal peptide is advantageous in promoting the dual transport of BKC-1 to the periplasm. This was reflected as lower MICs of ceftriaxone, cefotaxime, and ceftazidime in cells expressing BKC-1A compared to those that expressed BKC-1 (Table 4). Given that this duplication was reported in BKC-1 obtained from K. pneumoniae isolates and is not present in its proposed ancestral counterparts (72), this feature may be an evolutionary snapshot of an adaptation optimizing BKC-1 phenotypes in K. pneumoniae. Inexplicably, under β-lactam selection in the laboratory, we observed a propensity for the signal peptide coding region of the gene encoding BKC-1 to undergo in-frame triple-nucleotide insertions (data not shown), suggesting a mechanism by which this region can enhance the compatibility between BKC-1 and the new host E. coli. It was also shown very recently that BKC-1 was able to hydrolyze ceftazidime in E. coli, better than its putative *Shinella* counterpart enzymes (72), perhaps suggesting that this duplication might have occurred under selection in the more closely related Klebsiella, thereby enhancing the activity of BKC-1 against ceftazidime. Given that BKC-1 activity against ceftazidime in E. coli was abolished in the absence of TatC (Table 3 and 4), and otherwise greatly reduced for the other cephalosporins tested in E. coli, we suspect that BKC-1 could have adapted its signal peptide to confer a stronger resistance phenotype against ceftazidime and related cephalosporins. The observation of a similar phenotype with K. pneumoniae cells expressing the variant of BKC-1 that avoids the Tat translocon (Table 5) further supports this possibility.

The concept of dual targeting as exemplified by BKC-1 is further supported by observations of LGT-induced translocon switching for β-lactamases. For example, the β-lactamase BlaC from M. tuberculosis is exclusively translocated via the Tat system (48). When BlaC is expressed in E. coli, its translocation into the periplasm is exclusively via the Sec translocon (47). This speaks to the requirement of cytoplasmic folding in M. tuberculosis and further suggests that this particular polypeptide is prevented from folding in the cytoplasm of E. coli. The exact mechanism underpinning the ability of BKC-1 to interact with components of both the Tat and Sec pathways and the evolution of its unique signal peptide remains unknown, and understanding the folding intermediates of this dual translocon targeting enzyme would warrant further studies.

In principle, the carbapenem-resistance phenotype is readily transmissible on plasmids via LGT. Outbreaks of CRE having been reported in various countries (77–81) and CRE (K. pneumoniae) have become endemic in various parts of the world (22, 79, 81–83). While LGT is usually considered in terms of its benefits for the recipient, the recipient bacteria also suffer fitness costs with plasmid carriage because of the burdens associated with acquisition and maintenance of large quantities of DNA, as well as the need to correctly express, fold, and localize the “alien” proteins that these plasmids encode. In a given host scenario, the protein sequence features in a β-lactamase that increase protein folding efficiency do not necessarily correlate with enzyme efficiency and phenotype (84, 85), meaning the factors that drive the adaptability of β-lactamases in different species warrant further studies to better understand the spread of AMR phenotypes given current investments into next-generation antibiotics.

## MATERIALS AND METHODS

### Bacterial strains and plasmids.

Strains and plasmids used in this study are listed in Table S1 and Table S2, respectively, in the supplemental material. Plasmid construction details are described in Text S1. Primers used in this study are listed in Table S3. All cultures were grown in LB with the appropriate antibiotics at 37°C, shaking at 200 rpm unless otherwise mentioned (2.5 cm orbit). Growth curves were performed as described previously (67).

10.1128/mBio.01302-21.5 TABLE S1 List of strains used in this study. Download Table S1, PDF file, 0.2 MB. Copyright © 2021 Bharathwaj et al. 2021 Bharathwaj et al. https://creativecommons.org/licenses/by/4.0/ This content is distributed under the terms of the Creative Commons Attribution 4.0 International license.

10.1128/mBio.01302-21.6 TABLE S2 List of plasmids used in this study. Download Table S2, PDF file, 0.2 MB. Copyright © 2021 Bharathwaj et al. 2021 Bharathwaj et al. https://creativecommons.org/licenses/by/4.0/ This content is distributed under the terms of the Creative Commons Attribution 4.0 International license.

10.1128/mBio.01302-21.7 TABLE S3 List of primers and gBlocks used in this study. Download Table S3, PDF file, 0.1 MB. Copyright © 2021 Bharathwaj et al. 2021 Bharathwaj et al. https://creativecommons.org/licenses/by/4.0/ This content is distributed under the terms of the Creative Commons Attribution 4.0 International license.

### Protein expression, extraction, and analysis.

All strains containing pJPCmR and related vectors were grown to mid log-phase in LB supplemented with 34 μg/ml chloramphenicol and, if required, 35 ng/ml of anhydrotetracycline (ATc) for 4 h to induce protein expression. Crude cell lysates were prepared by harvesting 1 ml of cell culture and resuspending the pellets in an appropriate volume of SDS loading buffer (using a volume of 100 μl × the value of the optical density at 600 nm [OD_600_]). Periplasmic protein extracts were prepared using Tris-sucrose-EDTA (TSE) as described previously (86). Protein fractionation was performed as described previously (87). BL21 Star (DE3) cells harboring pETBKC-1 to express soluble cytoplasmic C-terminally hexahistidine-tagged BKC-1 were grown to an OD_600_ of 0.4 at 37°C in terrific broth (1.2% wt/vol tryptone, 2.4% wt/vol yeast extract, 0.4% vol/vol glycerol, 17 mM KH_2_PO_4_, and 72 mM K_2_HPO_4_) supplemented with 100 μg/ml ampicillin. BKC-1 expression was induced by adding isopropyl-β-d-thiogalactopyranoside (IPTG) to a final concentration of 0.2 mM and growing the culture for 4 h at 37°C. Cell lysis, protein extraction, purification, and quantification were performed as described previously (88). Using this protein, rabbit polyclonal antibodies against BKC-1 were obtained. SDS-PAGE and immunoblotting were performed as described previously (89) using the antibodies listed in Table S4.

10.1128/mBio.01302-21.8 TABLE S4 List of antibodies used in this study. Download Table S4, PDF file, 0.1 MB. Copyright © 2021 Bharathwaj et al. 2021 Bharathwaj et al. https://creativecommons.org/licenses/by/4.0/ This content is distributed under the terms of the Creative Commons Attribution 4.0 International license.

### Antibiotic sensitivity assays.

Cultures were grown to an OD_600_ of 0.6 to 0.7 and MICs were determined and analyzed using the broth microdilution method as outlined by CLSI (90). For cells that contained pJPCmR and its derivatives, cultures were grown to an OD_600_ of 0.3 to 0.4 and then induced with ATc at a final concentration of 35 ng/ml until an OD_600_ of 0.6 to 0.7 was reached before performing MIC assays in the continued presence of ATc.

### Sequence analysis.

Plasmid DNA comparison was performed using Easyfig 2.2.5 (91). Sequences of BKC-1 and other β-lactamases were obtained from BLDB (63). The sequence of BKC-1 was used as a query sequence to probe the database using the inbuilt BLDB BLAST function, and the top 200 hits were collected after setting a 40% sequence identity cutoff. Phylogeny trees were visualized using iTOL v4 (64) from alignments generated using Clustal Omega (92). Signal peptide analysis of BKC-1, BKC-1A, KPC-2, and L2 was performed using TatP 1.0 (93) and SignalP 5.0 (94) and all sequence alignments were visualized using ESPript (95).

10.1128/mBio.01302-21.1 TEXT S1 Plasmid construction. Text S1, DOCX file, 0.01 MB Copyright © 2021 Bharathwaj et al. 2021 Bharathwaj et al. https://creativecommons.org/licenses/by/4.0/ This content is distributed under the terms of the Creative Commons Attribution 4.0 International license.

10.1128/mBio.01302-21.9 TABLE S5 List of β-lactamases analyzed using iTOL. Download Table S5, PDF file, 0.5 MB. Copyright © 2021 Bharathwaj et al. 2021 Bharathwaj et al. https://creativecommons.org/licenses/by/4.0/ This content is distributed under the terms of the Creative Commons Attribution 4.0 International license.
