# Supplementary material for: The Carbapenemase BKC-1 from Klebsiella pneumoniae Is Adapted for Translocation by Both the Tat and Sec Translocons
Source: mBio. 2021 Jun 22;12(3):e01302-21. doi: 10.1128/mBio.01302-21 (PMC8262980; doi:10.1128/mBio.01302-21)
Supplement: FIG S1 [file mbio.01302-21-sf001.pdf]

```
             1        10        20        30        40        50        60        70        80
blaBKC-1     ATGACGATCACATTTTCGCGCCGGCAGGCGATTGCCGGCGCTCTCCTTGCCGTTCCCGCCGTGTCCACGCTGGCCGCCAG
blaGPC-1     ATGACGATAACCATTTTCACGCCGCCAG------------------------------------------------GCCAT
blaPAD-1     ATGACGATATCCCTTTCCCGCCGGCAG------------------------------------------------GCACT
            81        90       100       110       120       130       140       150       160
blaBKC-1     CGCCGGCGCTCTCCTTGCCGTTCCCGCCGTGTCCACGCTGGCCGCCAGCGCCGGGCGGCCACGGGCGGCCCGCTGGAAA
blaGPC-1     GGCCGGCGCTCTTCTTGCCATTCCCGCGGTTTCTGCGCTGACGGCCGGCACCAGCCGCGCCGCCGGTGAGAACCTTGCGC
blaPAD-1     TGCCGGAAGTCTGCTCGCCGTTCCGGCATTGGCCTCGCTGACAAGCGCCGCACGGGCGCAGAGCGGCGGCAAGCTGGAGA
           161       170       180       190       200       210       220       230       240
blaBKC-1     AGAGGCTGGCCGAGCTGGAAGGGCGTCACAAGGGCCGCATCGGCGTTGCCATACATAATCTCGCAACGGGCGCCCGCATC
blaGPC-1     AACGGCTTGCCGCATTAGAGGCACGCCACGGCGGGCGCATCGGCGTTGCAATACATAATCTTTCGACAGGCGCGCGGCTC
blaPAD-1     ACGAACTCGCAAAGCTTGAAGACAGGCATGGCGGCCGTATCGGCGTCGCGATCCTCAATCTCGCCACCGGCGACAAGATC
           241       250       260       270       280       290       300       310       320
blaBKC-1     GGCCACCGGGCGGACGAGCGCTTCCTGATGTGCAGCACCTTCAAGGCGTTGCTGGCGGCCCATATCCTCGCCCGCGTCGA
blaGPC-1     GGTCACAACACGGACGAGCGTTTCCTGATGTGCAGCACCTTCAAGGCGCTGCTGGCGGGCCATATCCTCGTCCGGGTGGA
blaPAD-1     GGACATCGTGCCGACGAACGCTTCCTGCTGTGCAGTACCTTCAAGGCCCTGACGGCAGGCTATATCCTCGCCCGCGTCGA
           321       330       340       350       360       370       380       390       400
blaBKC-1     CAGGAAGGAAGAAACGCTCGACCGGCGGATCGTCGTCGGGAAATCCGATCTTGTGGACTGGTCGCCGGTGGTGGAAACCC
blaGPC-1     CCGCGGCGAGGAGACGCTGGACCGACGCATCGTCGTGAAGGAAGCGGATCTCGTGGATTGGTCGCCGGTGGTGGAAAAGC
blaPAD-1     CAGAGGCGAGGAAGAGCTCGACCGGCGGATCGTCCATGCGAAGAAGGATCTGGTCACCTGGTCGCCGATCACGGAAAAGC
           401       410       420       430       440       450       460       470       480
blaBKC-1     GTGTCGGCGGCGAGGGCATCTCGATTGCCGAGCTTTGCGAGGCGGCGATCACGCTCAGCGACAACGCCGCGGCCAATCTC
blaGPC- 1    GCATCGGCGGGGATGTCATCTCTATTGCCGAACTCTGCGAGGCGACCATCACGCTCAGCGACAATGCCGCCGCCAACCTG
blaPAD-1     ACGTCGGCGGCGCCGGCATGTCCGTGGCCGAGCTCTGCCTGGCGACCGTGACCCTGAGCGACAACACCGCCGCCAATCTG
           481       490       500       510       520       530       540       550       560
blaBKC-1     CTGCTGTCCGCCTCGGGCGGGCCGAAGGCCGTCACGCAGTTCCTGCGCGGTTTCGGCGACGACGTGACGCGCCTCGACCG
blaGPC-1     CTGCTCGCCGCATCGGGCGGGCCTAAGGCCGTCACGGCGTTTCTGCGTGGCCTCGGCGATGAAGTGACGCGTCTCGACCG
blaPAD-1     CTGCTCAATAGCTTCGGCGGGCCGGCGGGCTGACGGCATTTTTGCGCTCCATCGGTGACGAGACCACGCGGCTCGACCG
           561       570       580       590       600       610       620       630       640
blaBKC-1     CACCGAACCGACGCTCAACTATCGCGAGACGCCGGACGACGAGCGCGACACGACCACGCCCGCCGCCATGGCCGAGACGC
blaGPC-1     TACGGAGCCGACGCTCAACTACCACGAGACGCCGGGCGATGAGCGTGACACGACGACGCCGAGCGCCATGGTGGAGACGC
blaPAD-1     TTTCGAAACCGAGCTGAATGTGCATGAAAAGCCGGGTGACCTGCGCGACACCACGACGCCCGCGGCCATGCTGGAGACGC
           641       650       660       670       680       690       700       710       720
blaBKC-1     TGCGCAAGCTGATCATCGGCGATGTCCTCGCGCGCGGCTCGAAGGCGCAGCTTGCCGCCTGGCTGGTGATGAACAAGACC
blaGPC-1     TGCGCAGGCTGCTCTTCACGGACGTTCTGTCGGCGCGTTCGAAGGCGCAGCTTGCCGCCTGGCTGATAATGAACAAGACG
blaPAD-1     TGCGCAAGCTTCTCTTCGGTGACGTGCTTTCCCGCTCCTCGCGCGCCCAGCTTGCCGCCTGGATGGTCATGAACAAAACG
           721       730       740       750       760       770       780       790       800
blaBKC-1     GGCGACACGCGATTGCGCGCCGGCTTCCCGGTCGACTGGACGACCGGCGACAAGACCGGCACGAACGGCGACAGGCACGG
blaGPC-1     GGCGACACGCGGCTGCGCGCTGGCTTCCCGGCAGACTGGATGACCGGCGACAAGACCGGCACCAACGGCGACAAGGCCGG
blaPAD-1     GGCGACGAAAAATTGCGGGCGGGCTTTCCCGCTGACTGGATGATCGGCGACAAGACCGGAGGCAATGGCGACAAATACGG
           801       810       820       830       840       850       860       870       880
blaBKC-1     CAACGCCAACGATGTCGCCATCGCCTGGTCGCCGGATCGCGGCGCGGTGGTCGTCACCGCTTTCTGCGAGATTCCCGGCA
blaGPC-1     AAACGCCAACGATGTCGCGGTCGCCTGGTCGCCGGATCGCGGTGCAGTCATTGTTGCCGCCTTCTGCGAAATCCCCGGCA
blaPAD-1     CAACAGTAACGACGTCGCGGTCGCCTGGTCACCGGACCGGGCGCCGTCATCGTGTCAGCCTATTGCGAGATACCGTCGG
           881       890       900       910       920       930       940
blaBKC-1     TTTCCGGGGATGAGCGCAACGCCGTCATCGCCGAGATTGGTCGCATTGCCGCCGAGGCCTGA
blaGPC-1     TTTCCGGTGACGAGCGCAATGCCGTTATTGCCGAAATCGGGCGCATCGCGGCAGAGGTCTGA
blaPAD-1     TCTCCGAAAAGGAGCGCGCCGCCATTCTGGCCGAAATCGGGCGGATCGCTTCGCGGGTCTAA
```

Figure S1. Alignment of *bla*BKC-1 with closely related genes. Nucleotide sequence alignment of *bla*BKC-1 from *K. pneumoniae*, *bla*GPC-1 from *P. aeruginosa*, and *bla*PAD-1 from *P. desertii*. Red highlight represents conserved nucleotides.
