# Supplementary material for: The Carbapenemase BKC-1 from Klebsiella pneumoniae Is Adapted for Translocation by Both the Tat and Sec Translocons
Source: mBio. 2021 Jun 22;12(3):e01302-21. doi: 10.1128/mBio.01302-21 (PMC8262980; doi:10.1128/mBio.01302-21)
Supplement: FIG S2 [file mbio.01302-21-sf002.pdf]

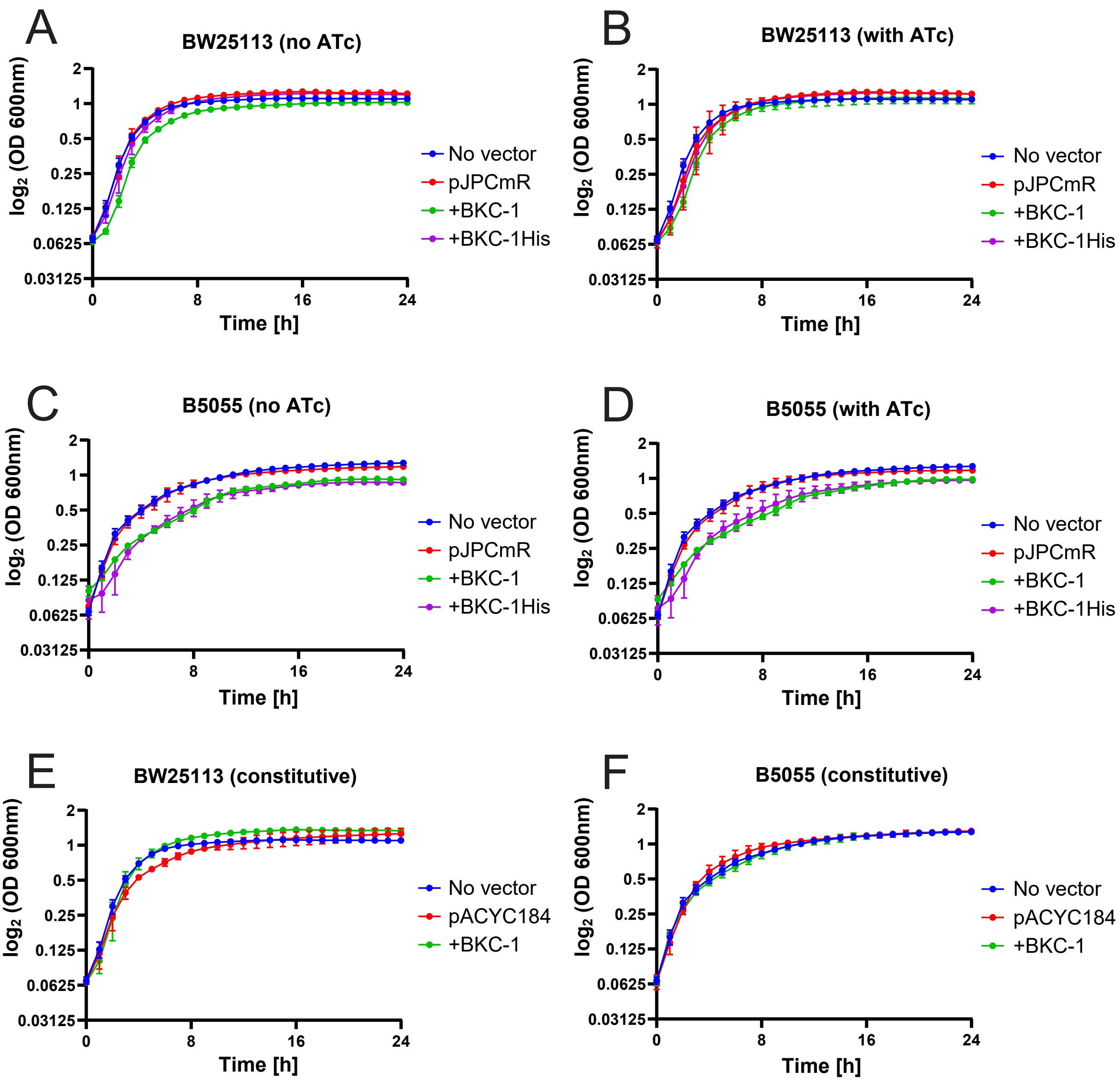

Figure S2. Growth curve analysis of *E. coli* and *K. pneumoniae* expressing *bla*$_{BKC-1}$. (A-B) Growth curves of *E. coli* BW25113 with inducible *bla*$_{BKC-1}$ constructs in the absence (A) and presence (B) of the inducer ATc. (C-D) Growth curves of *K. pneumoniae* B5055 with inducible *bla*$_{BKC-1}$ constructs in the absence (C) and presence (D) of the inducer ATc. (E-F) Growth curves of *E. coli* BW25113 (E) and *K. pneumoniae* B5055 (F) with constitutively expressing *bla*$_{BKC-1}$ constructs. (n=3, error bars represent standard deviations).
