# Supplementary material for: The Carbapenemase BKC-1 from Klebsiella pneumoniae Is Adapted for Translocation by Both the Tat and Sec Translocons
Source: mBio. 2021 Jun 22;12(3):e01302-21. doi: 10.1128/mBio.01302-21 (PMC8262980; doi:10.1128/mBio.01302-21)
Supplement: FIG S3 [file mbio.01302-21-sf003.pdf]

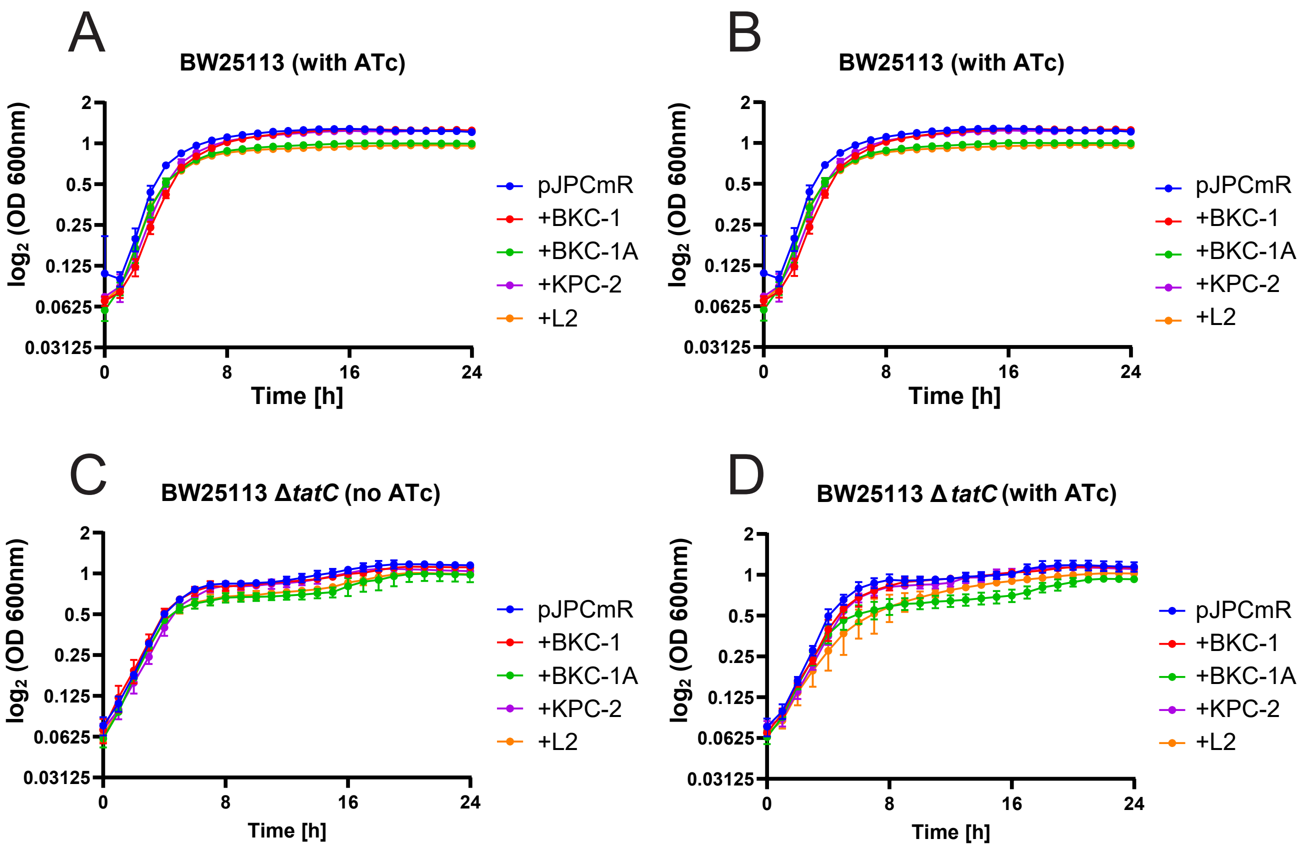

Figure S3. Growth curve analysis of *E. coli* expressing various β-lactamases. Growth curves of *E. coli* BW25113 (A-B) or its isogenic Δ*tatC* mutant (C-D) with inducible bla constructs in the absence (A, C) and presence (B, D) of the inducer ATc. The indicated β-lactamases contain a C-terminal His_6 tag. (n=3, error bars represent standard deviations).
