# Supplementary material for: The Carbapenemase BKC-1 from Klebsiella pneumoniae Is Adapted for Translocation by Both the Tat and Sec Translocons
Source: mBio. 2021 Jun 22;12(3):e01302-21. doi: 10.1128/mBio.01302-21 (PMC8262980; doi:10.1128/mBio.01302-21)
Supplement: TABLE S1 [file mbio.01302-21-st001.pdf]

TABLE S1 List of strains used in this study

Species	Strain	Properties	Reference
K. pneumoniae	B5055	K2:O1, mouse lethal clinical isolate	(1)
E. coli	BW25113 (wild type)	rrnB3 Δ*lac* Z4787 Δ*pho* BR580 hsdR514 Δ (*ara* BAD)567 Δ (*rha* BAD)568 *gal* U95 Δ*end* A9::FRT Δ*uid* A3::pir(wt) *recA* 1 *rph* -1	(2)
	BW25113 Δ*tatC* ::*kan*	Isogenic Δ*tatC* mutant of BW25113; kanamycin resistant	(3)
	BL21 Star™ (DE3)	fhuA2 *[lon] omp* T *gal (* λDE3*)* [dcm] Δ*hsd* S *λ DE3 = λ s* BamHI*o* Δ EcoRI-*B* int::(*lac* I*::* P*lac* UV5*::* T7 *gene1)* i21 Δ*nin* 5	Novagen
