# Supplementary material for: The Carbapenemase BKC-1 from Klebsiella pneumoniae Is Adapted for Translocation by Both the Tat and Sec Translocons
Source: mBio. 2021 Jun 22;12(3):e01302-21. doi: 10.1128/mBio.01302-21 (PMC8262980; doi:10.1128/mBio.01302-21)
Supplement: TABLE S2 [file mbio.01302-21-st002.pdf]

SUPPLEMENTARY TABLE S2 List of plasmids used in this study

Name	Replication	Resistance[1]	Characteristics	Source, Reference, or method of synthesis
pACYC184	p15A	Cm, Tc	Base vector	Chang & Cohen 1979
pACBKC	p15A	Cm, Amp	Constitutive *bla*$_{BKC-1}$ expression vector	pACYC184 (HindIII/SphI) Ω gBlock blaBKC-1_RBS_HindIII_SphI (HindIII/SphI; 972 bp)
pACYCDuet-1	p15A	Cm	Base vector	Novagen
pET-15b	ColE1	Amp	Base vector	Novagen
pETBKC-1	ColE1	Amp	IPTG inducible cytoplasmic bla$_{BKC-1}$ expression vector; C-terminal hexahistidine affinity tag	pET-15b (NcoI/NdeI) Ω BKC-1mat_NcoI_For/BKC-1_NdeI_Rev PCR product using pJPBKC-1His as template (NcoI/NdeI; 831 bp)
pJP-Cm	ColE1	Cm	Base vector	Rocker *et al.,* 2020
pJPBKC-1	ColE1	Cm, Amp*	ATc inducible *bla*$_{BKC-1}$ expression vector	pJP-Cm (EcoRI/HindIII) Ω gBlock blaBKC-1_EcoRI_HindIII (EcoRI/HindIII; 948 bp)
pJPBKC-1His	ColE1	Cm, Amp*	ATc inducible *bla*$_{BKC-1}$ expression vector; C-terminal hexahistidine affinity tag	pJP-Cm (EcoRI/HindIII) Ω gBlock blaBKC-1_His_EcoRI_HindIII (EcoRI/HindIII; 966 bp)
pJPBKC-1AHis	ColE1	Cm, Amp*	ATc inducible *bla*$_{BKC-1A}$ expression vector; C-terminal hexahistidine affinity tag	One-step Gibson Assembly of BKC-1HisGibsFor/BKC-1HisGibsRev PCR product using pJPBKC-1His template (DpnI treated; 4,772 bp)
pJPBKC-1KKHis	ColE1	Cm, Amp*	ATc inducible *bla*$_{BKC-1KK}$ expression vector; C-terminal hexahistidine affinity tag	pJP-Cm (EcoRI/HindIII) Ω BKC-1KK_EcoRI_For/BKC-1_HindIII_Rev PCR product using pJPBKC-1His as template (EcoRI/HindIII; 966bp)
pJPKPC-2His	ColE1	Cm, Amp*	ATc inducible *bla*$_{KPC-2}$ expression vector; C-terminal hexahistidine affinity tag	pJP-Cm (EcoRI/SphI) Ω KPC-2_EcoRI_For/KPC-2_His_SphI_Rev PCR product using pACDuetKPC-2 as template (EcoRI/SphI; 906 bp)
pJPL2His	ColE1	Cm, Amp*	ATc inducible *bla*$_{L2}$ expression vector; C-terminal hexahistidine affinity tag	pJP-Cm (EcoRI/HindIII) Ω L2_EcoRI_For/L2_His_HindIII_Rev PCR product using pACDuetL2 as template (EcoRI/HindIII; 936 bp)
pACDuetKPC-2	p15A	Cm, Amp*	IPTG inducible *bla*$_{KPC-2}$ expression vector	pACYCDuet-1 (NdeI/XhoI) Ω gBlock KPC-2 (NdeI/XhoI; 885 bp)
pACDuetL2	p15A	Cm, Amp*	IPTG inducible *bla*$_{L2}$ expression vector	pACYCDuet-1 (NdeI/XhoI) Ω gBlock L2 (NdeI/XhoI; 915 bp)

[1]Cm - Chloramphenicol resistance; Tc - Tetracycline resistance; Amp - Ampicillin resistance; Amp* - Inducible ampicillin resistance
