# Supplementary material for: The Carbapenemase BKC-1 from Klebsiella pneumoniae Is Adapted for Translocation by Both the Tat and Sec Translocons
Source: mBio. 2021 Jun 22;12(3):e01302-21. doi: 10.1128/mBio.01302-21 (PMC8262980; doi:10.1128/mBio.01302-21)
Supplement: TABLE S3 [file mbio.01302-21-st003.pdf]

SUPPLEMENTARY TABLE S3 List of primers and gBlocks used in this study

Primer name	Sequence (5' to 3')	Description
L2_EcoRI_For	TTACTTGAATTCATGTTGGCACGCCGTCGCTTTC	
L2_His_HindIII_Rev	TGCAAAGCTTTCAGTAATGATGATGATGATGCCCAATAAGGCGGTCTG	
KPC-2_EcoRI_For	CCTAGAATTCATGTCACTGTATCGCCGTCTAGTTCT	
KPC-2His_SphI_Rev	GTATGCATGCTTAATGATGATGATGATGATGCTGCCCGTTGACGCC	
BKC-1mat_NcoI_For	CTCGCCATGGATGGCTACTGGAGGGCCGCTGGAG	IPTG inducible expression of cytoplasmic BKC-1
BKC-1_NdeI_Rev	CAGCCATATGTCAATGATGATGATGATGATGGGCC	
BKC-1HisGibsFor	TGTCTACACTCGCTGCATCCGCAGGAGCAGCTACTGGAG	Removal of duplicated h region in BKC-1
BKC-1HisGibsRev	GGATGCAGCGAGTGTAGAC	
BKC-1KK_EcoRI_For	GGAGGAATTCATGACTATAACCTTCTCGAAGAAGCAAGCCATTG	
BKC-1_HindIII_Rev	AAGCTTTCAATGATGATGATGATGA	
Sequencing primers		
pJPMCS_For	CCTAATTTTTGTTGACACTCTATCATTG	Sequencing primer for pJP-Cm derived plasmids
pJPMCS_Rev	GCCAGGCAAATTCTGTTTTATCAGACCG	Sequencing primer for pJP-Cm derived plasmids
pACTetR_For	TCATGTTTGACAGCTTATCA	Sequencing primer for pACYC184 derived plasmids
pACTetR_Rev	TTTGCGCATTCACAGTTCTC	Sequencing primer for pACYC184 derived plasmids
T7promoter	TAATACGACTCACTATAGGG	Universal primer for sequencing pET-15b derived plasmids
T7terminator	GCTAGTTATTGCTCAGCGG	Universal primer for sequencing pET-15b derived plasmids

gBlocks (restriction sites designed for cloning purposes are shown in bold and underlined)

**Name**	**Sequence (5' to 3')**	**Description**
blaBKC-1_EcoRI_HindIII	CGCCCCGCGGCGTTGAATTCATGACTATAACCTTCTCGCGTCGTCAAG<br>CCATTGCAGGTGCGTTACTCGCCGTCCCTGCTGTGTCTACACTCGCTG<br>CATCCGCAGGTGCCTTGTTAGCGGTCCCGGCCGTCAGTACATTAGCTG<br>CCTCGGCAGGAGCAGCTACTGGAGGGCCGCTGGAGAAGCGGTTAGCCG<br>AACTTGAGGGCCGGCATAAAGGTCGTATTGGGGTGGCGATTCATAACC<br>TCGCCACCGGGGCCCGGATTGGACATCGGGCTGATGAGCGTTTCCTCA<br>TGTGCAGTACGTTCAAAGCTCTGCTCGCGGCGCATATTTTAGCACGTG<br>TCGATCGCAAAGAGGAGACCCTGGATCGCCGTATTGTGGTTGGGAAAT<br>CAGACTTAGTTGATTGGTCGCCTGTGGTAGAGACTCGTGTCGGGGGAG<br>AAGGGATCTCGATCGCGGAATTGTGTGAGGCGGCGATAACTCTCAGTG<br>ACAATGCTGCGGCCAATCTCCTCCTCAGTGCGAGTGGTGGTCCCAAAG<br>CGGTCACCCAATTCCTGCGTGGTTTCGGGGATGACGTCACTCGGCTTG<br>ATCGGACCGAACCAACGCTCAACTACCGTGAGACACCAGATGATGAGC<br>GGGACACCACTACACCTGCAGCAATGGCAGAGACGCTCCGGAAACTCA<br>TAATAGGGGATGTGTTGGCACGGGGATCGAAGGCGCAACTCGCGGCGT<br>GGTTGGTGATGAACAAGACAGGAGATACACGCCTTCGGGCAGGATTCC<br>CGGTCGACTGGACAACTGGTGATAAAACCGGCACCAATGGCGACCGGC<br>ATGGTAACGCCAACGACGTGGCCATTGCTTGGAGTCCTGATCGCGGCG<br>CAGTGGTTGTAACCGCCTTCTGTGAAATACCTGGAATCTCAGGGGACG<br>AGCGTAACGCAGTCATCGCTGAAATAGGGCGGATAGCAGCAGAGGCCT<br>GAAAGCTTCCGTATTTCCTGT	Sequence comprises a 14 bp clamping region, followed by *Eco*RI restriction site, then a 942 bp region derived from p60136 (Accession number: KP689347.1) comprising the bla_{BKC-1} gene (complement: 8,603-9,544) that had been partially codon optimised to remove regions of complexity. This is followed by the *Hin*dIII restriction site and a 13 bp clamping region
blaBKC-1_His_EcoRI_HindIII	CGCCCCGCGGCGTTGAATTCATGACTATAACCTTCTCGCGTCGTCAAG<br>CCATTGCAGGTGCGTTACTCGCCGTCCCTGCTGTGTCTACACTCGCTG<br>CATCCGCAGGTGCCTTGTTAGCGGTCCCGGCCGTCAGTACATTAGCTG<br>CCTCGGCAGGAGCAGCTACTGGAGGGCCGCTGGAGAAGCGGTTAGCCG<br>AACTTGAGGGCCGGCATAAAGGTCGTATTGGGGTGGCGATTCATAACC<br>TCGCCACCGGGGCCCGGATTGGACATCGGGCTGATGAGCGTTTCCTCA<br>TGTGCAGTACGTTCAAAGCTCTGCTCGCGGCGCATATTTTAGCACGTG<br>TCGATCGCAAAGAGGAGACCCTGGATCGCCGTATTGTGGTTGGGAAAT<br>CAGACTTAGTTGATTGGTCGCCTGTGGTAGAGACTCGTGTCGGGGGAG<br>AAGGGATCTCGATCGCGGAATTGTGTGAGGCGGCGATAACTCTCAGTG<br>ACAATGCTGCGGCCAATCTCCTCCTCAGTGCGAGTGGTGGTCCCAAAG<br>CGGTCACCCAATTCCTGCGTGGTTTCGGGGATGACGTCACTCGGCTTG<br>ATCGGACCGAACCAACGCTCAACTACCGTGAGACACCAGATGATGAGC<br>GGGACACCACTACACCTGCAGCAATGGCAGAGACGCTCCGGAAACTCA<br>TAATAGGGGATGTGTTGGCACGGGGATCGAAGGCGCAACTCGCGGCGT<br>GGTTGGTGATGAACAAGACAGGAGATACACGCCTTCGGGCAGGATTCC<br>CGGTCGACTGGACAACTGGTGATAAAACCGGCACCAATGGCGACCGGC<br>ATGGTAACGCCAACGACGTGGCCATTGCTTGGAGTCCTGATCGCGGCG<br>CAGTGGTTGTAACCGCCTTCTGTGAAATACCTGGAATCTCAGGGGACG<br>AGCGTAACGCAGTCATCGCTGAAATAGGGCGGATAGCAGCAGAGGCCC<br>ATCATCATCATCATCATTGAAAGCTTCCGTATTTCCTGT	Sequence comprises a 14 bp clamping region, followed by *Eco*RI restriction site, then a 942 bp region derived from p60136 (Accession number: KP689347.1) comprising the bla_{BKC-1} gene (complement: 8,603-9,544) that had been partially codon optimised to remove regions of complexity. A series of 18 bp were inserted immediately before the stop codon encoding the hexahistidine tag. This is followed by the *Hind*III restriction site and a 13 bp clamping region

SUPPLEMENTARY TABLE S3 List of primers and gBlocks used in this study (continued)

gBlocks (restriction sites designed for cloning purposes are shown in bold and underlined)		
**Name**	**Sequence (5’ to 3’)**	**Description**
blaBKC-1_RBS_HindIII_SphI	CCGCGGCGTTAAGCTTTAAACAGACACAGGAAAAATACGGATGACTATAAC<br>CTTCTCGCGTCGTCAAGCCATTGCAGGTGCGTTACTCGCCGTCCCTGCTGT<br>GTCTACACTCGCTGCATCCGCAGGTGCCTTGTTAGCGGTCCCGGCCGTCAG<br>TACATTAGCTGCCTCGGCAGGAGCAGCTACTGGAGGGCCGCTGGAGAAGCG<br>GTTAGCCGAACTTGAGGGCCGGCATAAAGGTCGTATTGGGGTGGCGATTCA<br>TAACCTCGCCACCGGGGCCCGGATTGGACATCGGGCTGATGAGCGTTTCCT<br>CATGTGCAGTACGTTCAAAGCTCTGCTCGCGGCGCATATTTTAGCACGTGT<br>CGATCGCAAAGAGGAGACCCTGGATCGCCGTATTGTGGTTGGGAAATCAGA<br>CTTAGTTGATTGGTCGCCTGTGGTAGAGACTCGTGTCGGGGGAGAAGGGAT<br>CTCGATCGCGGAATTGTGTGAGGCGGCGATAACTCTCAGTGACAATGCTGC<br>GGCCAATCTCCTCCTCAGTGCGAGTGGTGGTCCCAAAGCGGTCACCCAATT<br>CCTGCGTGGTTTCGGGGATGACGTCACTCGGCTTGATCGGACCGAACCAAC<br>GCTCAACTACCGTGAGACACCAGATGATGAGCGGGACACCACTACACCTGC<br>AGCAATGGCAGAGACGCTCCGGAAACTCATAATAGGGGATGTGTTGGCACG<br>GGGATCGAAGGCGCAACTCGCGGCGTGGTTGGTGATGAACAAGACAGGAGA<br>TACACGCCTTCGGGCAGGATTCCCGGTCGACTGGACAACTGGTGATAAAAC<br>CGGCACCAATGGCGACCGGCATGGTAACGCCAACGACGTGGCCATTGCTTG<br>GAGTCCTGATCGCGGCGCAGTGGTTGTAACCGCCTTCTGTGAAATACCTGG<br>AATCTCAGGGGACGAGCGTAACGCAGTCATCGCTGAAATAGGGCGGATAGC<br>AGCAGAGGCCTGAGCATGCCCGTATTTC	Sequence comprises a 10 bp clamping region, followed by *Hin*dIII restriction site, then a 966 bp region derived from p60136 (Accession number: KP689347.1) comprising a codon optimised bla_{BKC-1} gene (complement: 8,603-9,544) with its unmodified upstream region (coloured red; complement: 9,545-9,568) that includes the native RBS. This is followed by the *Sph*I restriction site and another 10 bp clamping region
KPC2	GATATACATATGTCACTGTATCGCCGTCTAGTTCTGCTGTCTTGTCTCTCA<br>TGGCCGCTGGCTGGCTTTTCTGCCACCGCGCTGACCAACCTCGTCGCGGAA<br>CCATTCGCTAAACTCGAACAGGACTTTGGCGGCTCCATCGGTGTGTACGCG<br>ATGGATACCGGCTCAGGCGCAACTGTAAGTTACCGCGCTGAGGAGCGCTTC<br>CCACTGTGCAGCTCATTCAAGGGCTTTCTTGCTGCCGCTGTGCTGGCTCGC<br>AGCCAGCAGCAGGCCGGCTTGCTGGACACACCCATCCGTTACGGCAAAAAT<br>GCGCTGGTTCCGTGGTCACCCATCTCGGAAAAATATCTGACAACAGGCATG<br>ACGGTGGCGGAGCTGTCCGCGGCCGCCGTGCAATACAGTGATAACGCCGCC<br>GCCAATTTGTTGCTGAAGGAGTTGGGCGGCCCGGCCGGGCTGACGGCCTTC<br>ATGCGCTCTATCGGCGATACCACGTTCCGTCTGGACCGCTGGGAGCTGGAG<br>CTGAACTCCGCCATCCCAGGCGATGCGCGCGATACCTCATCGCCGCGCGCC<br>GTGACGGAAAGCTTACAAAAACTGACACTGGGCTCTGCACTGGCTGCGCCG<br>CAGCGGCAGCAGTTTGTTGATTGGCTAAAGGGAAACACGACCGGCAACCAC<br>CGCATCCGCGCGGCGGTGCCGGCAGACTGGGCAGTCGGAGACAAAACCGGA<br>ACCTGCGGAGTGTATGGCACGGCAAATGACTATGCCGTCGTCTGGCCCACT<br>GGGCGCGCACCTATTGTGTTGGCCGTCTACACCCGGGCGCCTAACAAGGAT<br>GACAAGCACAGCGAGGCCGTCATCGCCGCTGCGGCTAGACTCGCGCTGGAA<br>GGATTGGGCGTCAACGGGCAGTAACTCGAGATGC	Sequence comprises a 6 bp clamping region, followed by an *Nde*I restriction site, then an 879 bp region derived from pKP05 (Accession number: MK330868.1), comprising the native bla_{KPC-2} gene (8,671-9,549) with the internal (and in-frame) *Xho*I restriction site CTCGAG (9,520-9,525) removed by codon optimisation (converted to CTGGAA). This is followed by an *Xho*I restriction site and a 4 bp clamping region. NB: This gene will use the internal start codon from the *Nde*I restriction site.
L2	GATATACATATGTTGGCACGCCGTCGCTTTCTGCAATTTTCAGGTGCCGCC<br>GTTGCCTCCTCTCTTGCCTTACCGCTGTTGGCCCGTGCAGCGGGCAAGGCC<br>ACGGCAAACGCGCCCACAGACGCGGCGATTACTGCAGCCTCCGATTTCGCT<br>GCTTTAGAGAAGGCTTGCGCCGGACGCCTTGGGGTTACTTTACTGGATACA<br>GCGTCTGGCCGCCGCATCGGGCACCGTCAGGACGAACGTTTCCCGATGTGT<br>TCGACATTCAAAAGTATGCTTGCAGCAACAGTGCTTAGTCAAGCAGAACGT<br>ATGCCAGCACTTTTAGACCGTCGCGTGCCGGTCGGCGAGGCGGACTTACTG<br>AGCCATGCACCTGTCACTCGCCGTCACGCAGGGAAGGATATGACGGTGCGC<br>GATTTGTGCCGTGCAACAATCATTACGTCCGATAACACCGCAGCCAATTTG<br>TTGTTTGGGGTGGTGGGGGGTCCTCCAGCGGTGACTGCTTTCTTACGCGCC<br>TCGGGCGATACGGTTTCGCGTTCAGATCGTTTGGAACCCGAGTTAAACAGT<br>TTCGCTAAAGGCGACCCACGTGATACCACAACTCCTGCAGCGATGGCCGCA<br>ACGTTACAGCGCGTTGTACTGGGAGAGGTTTTGCAGCCCGCCTCTCGTCAG<br>CAGTTAGCTGATTGGTTAATTGACAACGAGACCGGCGACGCATGTTTGCGC<br>GCCGGCCTTGGTAAACGTTGGCGCGTGGGCGATAAAACGGGTTCCAATGGC<br>GAGGACGCACGCAACGACATCGCCGTGTTATGGCCAGTGGCTGGCGGAGCA<br>CCCTGGGTACTGACAGCCTACCTTCAGGCGGGTGCTATCAGCTATGAACAA<br>CGTGCATCCGTACTGGCGCAAGTAGGTCGTATTGCAGACCGCCTTATTGGG<br>TGACTCGAGATGC	Sequence comprises a 6 bp clamping region, followed by an *Nde*I restriction site, then a 909 bp region derived from a *Stenotrophomonas maltophilia* K279a plasmid, comprising the bla_{L2b} (Accession: AJ251816.1: 4-912) that had been partially codon optimised to remove regions of complexity. This is followed by an *Xho*I restriction site and a 4 bp clamping region. NB: This gene will use the internal(and in-frame) start codon from the *Nde*I restriction site.
