# Supplementary material for: The Carbapenemase BKC-1 from Klebsiella pneumoniae Is Adapted for Translocation by Both the Tat and Sec Translocons
Source: mBio. 2021 Jun 22;12(3):e01302-21. doi: 10.1128/mBio.01302-21 (PMC8262980; doi:10.1128/mBio.01302-21)
Supplement: TABLE S4 [file mbio.01302-21-st004.pdf]

TABLE S4 List of antibodies used in this study

Name of antibody	Type	Epitope source and host	Dilution	Host	Source
α-BKC-1	Polyclonal	Raised against mature BKC-1 expressed in *E. coli*	1:20,000 (2% milk in TBST)	Rabbit	This study
α-HisHRP	Monoclonal, Conjugated (one-step)	Raised against hexahistidine and mouse IgG1	1:20,000 (2% milk in TBST)	Mouse	R&D Systems MAB050H
α-SurA	Polyclonal	Raised against *E. coli* SurA	1:30,000 (2% milk in TBST)	Rabbit	Laboratory stock
α-TatC	Polyclonal	Raised against *E. coli* TatC	1:10,000 (TBST)	Rabbit	Gifted by Tracy Palmer, Newcastle University
α-BamA	Polyclonal	Raised against *E. coli* BamA	1:20,000 (2% milk in TBST)	Rabbit	Laboratory stock
α-$F_1\beta$	Polyclonal	Raised against $F_1\beta$ subunit of the ATPase purified from *Saccharomyces cerevisiae*	1:4000 (2% milk in TBST)	Rabbit	Laboratory stock
Goat α-rabbit -IgG	Polyclonal	Rabbit IgG	1:20,000 (2% milk in TBST)	Goat	ThermoFisher® A21245
