# Supplementary material for: The Carbapenemase BKC-1 from Klebsiella pneumoniae Is Adapted for Translocation by Both the Tat and Sec Translocons
Source: mBio. 2021 Jun 22;12(3):e01302-21. doi: 10.1128/mBio.01302-21 (PMC8262980; doi:10.1128/mBio.01302-21)
Supplement: TEXT S1 [file mbio.01302-21-s0001.docx]

**SUPPLEMENTARY METHODS**

**Plasmid construction**

Plasmids are listed in Table S2. Primers and gBlocks used to generate (or confirm) plasmids are listed in Table S3. To generate the constitutively expressing *bla*_BKC-1_ construct, pACBKC, the blaBKC-1_RBS_HindIII_SphI gBlock was cloned in to the *Hin*dIII/*Sph*I site of pACYC184. To generate the ATc inducible *bla*_BKC-1_ construct, pJPBKC-1, the blaBKC-1_His_EcoRI_HindIII gBlock was cloned in to the *Eco*RI/*Hin*dIII site of pJPCmR

To generate the C-terminally His_6_ tagged ATc inducible *bla*_BKC-1_ construct, pJPBKC-1His, the blaBKC-1_His_EcoRI_HindIII gBlock was cloned in to the *Eco*RI/*Hin*dIII site of pJPCmR. To remove the 16-residue duplication in the signal sequence of BKC-1, to create BKC-1A, a fragment from pJPBKC-1His (4,781 bp) was amplified with BKC-1HisGibsFor and BKC-1HisGibsRev primers that would exclude the 48 bp region corresponding to the duplicated sequence. The PCR product was treated with *Dpn*I to remove methylated template DNA and the assembly of the remaining product was performed using Gibson Assembly Master Mix (NEB). This construct was called pJPBKC-1AHis. As part of another ongoing project, pACDuetKPC-2 and pACDuetL2 were generated by cloning the KPC-2 or L2 gBlock, respectively, in to the *Nde*I/*Xho*I site of pACYCDuet-1. These constructs were subsequently used as a template to generate the C-terminally His_6_ tagged β-lactamase constructs pJPKPC-2His and pJPL2His, respectively. To generate pJPKPC-2His, a 906 bp PCR product was amplified using the primer pair KPC-2_EcoRI_For and KPC-2_His_SphI_Rev and cloned in to the *Eco*RI/*Sph*I site of pJPCmR. To generate pJPL2His, a 936 bp PCR product was amplified using the primer pair L2_EcoRI_For and L2-His_HindIII_Rev and cloned in to the *Eco*RI/*Hin*dIII site of pJPCmR. To generate pJPBKC-1KKHis, a 976bp PCR product was amplified using the primer pair BKC-1KK_EcoRI_For and BKC-1_HindIII_Rev and cloned into the *Eco*RI/*Hin*dIII site of pJPCmR. To generate the C-terminally His_6_ tagged IPTG-inducible *bla*_BKC-1_, pETBKC-1, an 831 bp PCR product was amplified using the primer pair BKC-1mat_NcoI_For and BKC-1_NdeI_Rev with pJPBKC-1His as template and cloned in to the *Nco*I/*Nde*I site of pET-15b. The β-lactamase synthesised using this construct is localised to the cytoplasm (because it does not contain a signal peptide) and was used to purify mature BKC-1 for antibody production.

**SUPPLEMENTARY REFERENCES**

1. Clements A, Tull D, Jenney AW, Farn JL, Kim SH, Bishop RE, McPhee JB, Hancock RE, Hartland EL, Pearse MJ, Wijburg OL, Jackson DC, McConville MJ, Strugnell RA. 2007. Secondary acylation of *Klebsiella pneumoniae* lipopolysaccharide contributes to sensitivity to antibacterial peptides. J Biol Chem 282:15569-77.

2. Lessard IA, Pratt SD, McCafferty DG, Bussiere DE, Hutchins C, Wanner BL, Katz L, Walsh CT. 1998. Homologs of the vancomycin resistance d-Ala-d-Ala dipeptidase VanX in *Streptomyces toyocaensis*, *Escherichia coli* and *Synechocystis*: Attributes of catalytic efficiency, stereoselectivity and regulation with implications for function. Chem Biol 5:489-504.

3. Baba T, Ara T, Hasegawa M, Takai Y, Okumura Y, Baba M, Datsenko KA, Tomita M, Wanner BL, Mori H. 2006. Construction of *Escherichia coli* K-12 in-frame, single-gene knockout mutants: The Keio collection. Mol Syst Biol 2:2006.0008.

4. Chang AC, Cohen SN. 1978. Construction and characterization of amplifiable multicopy DNA cloning vehicles derived from the P15A cryptic miniplasmid. J Bacteriol 134:1141-56.

5. Rocker A, Lacey JA, Belousoff MJ, Wilksch JJ, Strugnell RA, Davies MR, Lithgow T. 2020. Global trends in proteome remodeling of the outer membrane modulate antimicrobial permeability in *Klebsiella pneumoniae*. mBio 11.
