# Supplementary material for: The Carbapenemase BKC-1 from Klebsiella pneumoniae Is Adapted for Translocation by Both the Tat and Sec Translocons
Source: mBio. 2021 Jun 22;12(3):e01302-21. doi: 10.1128/mBio.01302-21 (PMC8262980; doi:10.1128/mBio.01302-21)
Supplement: TABLE S5 [file mbio.01302-21-st005.pdf]

TABLE S5 List of β-lactamases analysed using iTOL

Protein name	Organism	Accession
BKC-1	*Klebsiella pneumoniae*	AKD43328.1
GPC-1	*Pseudomonas aeruginosa*	AWB15813.1
PAD-1	*Paramesorhizobium deserti*	KXF74838.1
AXC-2	*Achromobacter ruhlandii*	AXM44138.1
AXC-3	*Achromobacter ruhlandii*	AXM44139.1
BPS-7	*Burkholderia pseudomallei*	AAK52328.1
BPS-5	*Burkholderia oklahomensis*	AIO70431.1
AXC-4	*Achromobacter xylosoxidans*	QEJ74006.1
AXC-1	*Achromobacter xylosoxidans*	ATG32091.1
BPS-1b	*Burkholderia pseudomallei*	AAL35334.1
BPS-1	*Burkholderia pseudomallei*	AAK37506.1
BPS-1d	*Burkholderia pseudomallei*	AAL35336.1
BPS-2	*Burkholderia pseudomallei*	AAK52327.1
BPS-1m	*Burkholderia pseudomallei*	AAM10778.1
AXC-5	*Achromobacter xylosoxidans*	QEJ80833.1
PAU-1	*Pseudomonas aeruginosa*	APC57487.2
BPS-1c	*Burkholderia pseudomallei*	AAL35335.1
BOR-1	*Bordetella bronchiseptica*	CAE32545.1
BPS-6	*Burkholderia pseudomallei*	AAK52324.1
PenB-5	*Burkholderia cepacia*	AIO27915.1
BPS-3	*Burkholderia thailandensis*	AJY01305.1
BPS-4	*Burkholderia sp*	AJY38930.1
CARB-5	*Acinetobacter calcoaceticus*	AAF61417.1
CARB-16	*Acinetobacter pittii*	OCY97266.1
CARB-14	*Acinetobacter baumannii*	AFI72872.1
CARB-8	*Oligella urethralis*	AAO59455.1
CARB-49	*Acinetobacter baumannii*	ANW47155.1
CzoA-1	*Comamonas testosteroni*	EFI60385.1
CARB-50	*Acinetobacter baumannii*	AXH79875.1
CTX-M-123	*Escherichia coli*	AFA51701.1
CARB-10	*Acinetobacter baumannii*	ACJ61335.1
LUT-3	*Pseudomonas luteola*	ACN76563.1
LUT-4	*Pseudomonas luteola*	ACN76564.1
LUT-1	*Pseudomonas luteola*	AAU10324.1
LUT-2	*Pseudomonas luteola*	ACN76562.1
KLUC-3	*Escherichia coli*	AFQ36037.1
LUT-5	*Pseudomonas luteola*	ACN76565.1
OXY-2-9	*Klebsiella oxytoca*	ACV44455.1
CTX-M-P6	*Shigella spp*	AEM44654.1
CTX-M-33	*Escherichia coli*	AAO88912.1
FEC-1	*Escherichia coli*	BAC53608.1

TABLE S5 List of β-lactamases analysed using iTOL (continued)

Protein name	Organism	Accession
CTX-M-182	*Escherichia coli*	AMY95408.1
CTX-M-186	*Escherichia coli*	ANN89336.1
LUT-6	*Pseudomonas luteola*	ACN76566.1
CTX-M-170	*Escherichia coli*	ALK03059.1
CTX-M-88	*Salmonella spp*	ACP18863.1
CTX-M-216	*Escherichia coli*	AWI33306.1
CTX-M-142	*Escherichia coli*	AGW25368.1
KLUC-5	*Raoultella ornithinolytica*	PJR05208.1
CTX-M-156	*Klebsiella pneumoniae*	AIS67612.1
CTX-M-139	*Escherichia coli*	AFY98865.1
CTX-M-220	*Klebsiella pneumoniae*	AWU66425.1
OXY-2-3	*Klebsiella oxytoca*	AAL78281.2
CTX-M-163	*Escherichia coli*	AKO63214.1
CTX-M-228	*Enterobacter cloacae*	QDY98372.1
CTX-M-225	*Escherichia coli*	QBO66647.1
CTX-M-193	*Escherichia coli*	AQM40188.1
CTX-M-173	*Klebsiella pneumoniae*	ALM96711.1
CTX-M-224	*Enterobacter kobei*	AYJ76543.1
CTX-M-169	*Escherichia coli*	ALL29307.1
CTX-M-114	*Providencia rettgeri*	ACU00153.1
CTX-M-15	*Acinetobacter baumannii*	AEQ20893.1
CTX-M-188	*Escherichia coli*	AOA70434.1
CTX-M-28	*Escherichia coli*	ABS12043.1
CTX-M-194	*Escherichia coli*	AQM40189.1
CTX-M-96	*Enterobacter hormaechei*	KTK23935.1
L2-20	*Stenotrophomonas maltophilia*	WP_049415333.1
CTX-M-101	*Escherichia coli*	ADY02545.1
OXY-2-4	*Klebsiella oxytoca*	AAL78280.2
CTX-M-29	*Escherichia coli*	AAP22736.1
CTX-M-176	*Klebsiella pneumoniae*	ALM96715.1
KLUC-1	*Kluyvera cryocrescens*	AAK08976.1
CTX-M-197	*Klebsiella pneumoniae*	AQT03461.1
CTX-M-82	*Escherichia coli*	ABB59946.1
CTX-M-209	*Klebsiella pneumoniae*	AVP74334.1
CTX-M-202	*Escherichia coli*	ARX71248.1
CTX-M-208	*Klebsiella pneumoniae*	AVP74333.1
CTX-M-183	*Klebsiella pneumoniae*	ANH52938.1
CTX-M-71	*Klebsiella pneumoniae*	ACV92002.1
CTX-M-132	*Escherichia coli*	AFQ94051.1
CTX-M-103	*Escherichia coli*	CDG50843.1
CTX-M-184	*Escherichia coli*	ANH52939.1

TABLE S5 List of β-lactamases analysed using iTOL (continued)

Protein name	Organism	Accession
CTX-M-226	*Escherichia coli*	QBX33278.1
CTX-M-36	*Enterobacter cloacae*	SAD14715.1
CTX-M-32	*Escherichia coli*	AOG15804.1
CTX-M-162	*Klebsiella oxytoca*	AKO63213.1
CTX-M-206	*Unknown*	AIC64344.1
CTX-M-211	*Escherichia coli*	AVP74336.1
CTX-M-P4	*Shigella spp*	AEM44650.1
CTX-M-232	*Escherichia coli*	QFR38189.1
CTX-M-210	*Escherichia coli*	AVP74335.1
CTX-M-218	*Escherichia coli*	AWI33312.1
CTX-M-68	*Klebsiella*	ABV81082.1
CTX-M-69	*Escherichia coli*	ABY91281.1
CTX-M-22	*Enterobacter cloacae*	AAX58181.1
KLUB-1	*Aeromonas hydrophila*	AFI26272.1
CTX-M-230	*Escherichia coli*	QFR38187.1
CTX-M-30	*Citrobacter freundii*	AAP43508.1
CTX-M-231	*Klebsiella pneumoniae*	QFR38188.1
CTX-M-12	*Enterobacter hormaechei*	KJL64335.1
CTX-M-154	*Klebsiella pneumoniae*	PNS00289.1
L2-28	*Stenotrophomonas maltophilia*	ALA83631.1
CTX-M-164	*Proteus mirabilis*	AKR53959.1
CTX-M-79	*Escherichia coli*	ABO09821.1
CTX-M-55	*Escherichia coli*	ABI34705.1
CTX-M-P5	*Shigella spp*	AEM44651.1
CTX-M-10	*Escherichia coli*	AAF65843.1
CTX-M-180	*Escherichia coli*	AMY95406.1
CTX-M-178	*Escherichia coli*	AMB17206.1
CTX-M-61	*Salmonella spp*	ABN09669.1
L2-79	*Stenotrophomonas maltophilia*	QCX20020.1
CTX-M-1	*Escherichia coli*	ABK06383.1
CTX-M-189	*Escherichia coli*	ACN39712.1
CTX-M-143	*Escherichia coli*	QGN18754.1
CTX-M-127	*Escherichia coli*	ARX70572.1
CTX-M-181	*Escherichia coli*	AMY95407.1
CTX-M-144	*Escherichia coli*	AHX39588.1
CTX-M-177	*Enterobacter cloacae*	ALM96716.1
CTX-M-53	*Salmonella spp*	ABB72225.1
CTX-M-66	*Proteus mirabilis*	ABQ45409.1
KLUC-4	*Enterobacter cloacae*	AFQ36038.1
CTX-M-222	*Escherichia coli*	AXI82473.1
CTX-M-80	*Klebsiella pneumoniae*	ABW86620.2

TABLE S5 List of β-lactamases analysed using iTOL (continued)

Protein name	Organism	Accession
CTX-M-158	*Escherichia coli*	AIT97310.1
KLUB-2	*Enterobacter cloacae*	AAT70415.1
CTX-M-157	*Klebsiella pneumoniae*	AIS67613.1
CTX-M-203	*Escherichia coli*	ARX70573.1
CTX-M-172	*Escherichia coli*	AKS25179.1
KLUC-2	*Enterobacter cloacae*	ABM73648.1
CTX-M-60	*Klebsiella pneumoniae*	CAL80726.1
CTX-M-116	*Proteus mirabilis*	AEI70324.1
CTX-M-136	*Escherichia coli*	AMP17732.1
CTX-M-190	*Escherichia coli*	AOF41435.1
CTX-M-187	*Enterobacter cloacae*	ANY40236.1
CTX-M-64	*Enterobacter cloacae*	ACU87985.1
OXY-2-6	*Klebsiella oxytoca*	AAL78278.2
OXY-2-14	*Klebsiella oxytoca*	AAQ75378.1
CTX-M-167	*Proteus mirabilis*	ALJ77593.1
CTX-M-204	*Klebsiella pneumoniae*	ARX71259.1
CTX-M-146	*Escherichia coli*	ARI46098.1
CTX-M-207	*Escherichia coli*	BBC20336.1
OXY-2-19	*Klebsiella oxytoca*	ALC79262.1
CTX-M-117	*Escherichia coli*	AET99223.1
CTX-M-166	*Escherichia coli*	ANA76436.1
CTX-M-227	*Escherichia coli*	QDM39442.1
CTX-M-175	*Escherichia coli*	ALM96714.1
CTX-M-155	*Klebsiella pneumoniae*	AIS67611.1
CTX-M-138	*Escherichia coli*	AHA80107.1
CTX-M-34	*Escherichia coli*	AAR99493.1
OXY-1-4	*Klebsiella oxytoca*	AAL78276.1
CTX-M-199	*Escherichia coli*	ARA73610.1
L2-3	*Stenotrophomonas maltophilia*	CAB63491.1
OXY-1-8	*Klebsiella oxytoca*	EWF75334.1
OXY-2-7	*Klebsiella oxytoca*	AFV73218.1
CTX-M-150	*Escherichia coli*	AHF20911.1
CTX-M-62	*Klebsiella pneumoniae*	ABP04245.1
OXY-2-2	*Klebsiella oxytoca*	AAL79541.1
CTX-M-72	*Klebsiella pneumoniae*	AAV97957.1
PenB-2	*Burkholderia cenocepacia*	ACO36249.1
CTX-M-54	*Klebsiella pneumoniae*	ABC18328.3
CTX-M-42	*Escherichia coli*	AAY84742.1
CTX-M-179	*Escherichia coli*	AMJ17432.1
OXY-2-12	*Klebsiella oxytoca*	AFV73217.1
OXY-2-11	*Klebsiella oxytoca*	AAL78282.3

TABLE S5 List of β-lactamases analysed using iTOL (continued)

Protein name	Organism	Accession
CTX-M-58	*Escherichia coli*	ABM97538.1
OXY-6-2	*Klebsiella oxytoca*	BAS39381.1
OXY-2-1	*Klebsiella oxytoca*	CAI43414.1
OXY-2-10	*Klebsiella oxytoca*	ACV44456.1
CTX-M-52	*Klebsiella pneumoniae*	ABB17185.1
OXY-1-2	*Klebsiella oxytoca*	AAL78277.1
OXY-2-8	*Klebsiella oxytoca*	AAL17873.1
OXY-1-3	*Klebsiella oxytoca*	AAL78275.1
OXY-2-13	*Klebsiella oxytoca*	AFV73219.1
CTX-M-23	*Escherichia coli*	AAL99990.1
CTX-M-195	*Escherichia coli*	AQM40190.1
CTX-M-212	*Proteus mirabilis*	AVP74337.1
OXY-2-20	*Klebsiella oxytoca*	ALC79263.1
OXY-2-17	*Klebsiella oxytoca*	ALC79260.1
OXY-1-7	*Klebsiella oxytoca*	AWG41961.1
PenA	*Burkholderia cepacia*	AAB53622.1
OXY-1-1	*Klebsiella oxytoca*	AEX04382.1
OXY-2-b	*Klebsiella oxytoca*	AAQ75379.1
PenB-4	*Burkholderia cenocepacia*	ACO36252.1
OXY-2-18	*Klebsiella oxytoca*	ALC79261.1
CTX-M-38	*Klebsiella pneumoniae*	AAV70602.1
XCC-16	*Xanthomonas campestris*	RFF52301.1
PenB-3	*Burkholderia cenocepacia*	ACO36251.1
CTX-M-P44	*Klebsiella pneumoniae*	AAF93177.1
PenB-1	*Burkholderia cenocepacia*	ACJ63454.1
CTX-M-121	*Escherichia coli*	AFA51699.1
OXY-1-6	*Klebsiella oxytoca*	CAB42615.1
CTX-M-84	*Salmonella spp*	ACI29346.1
CTX-M-16	*Escherichia coli*	AAK32961.1
CTX-M-27	*Escherichia coli*	AAO61597.1
OXY-2-16	*Klebsiella oxytoca*	ESM76803.1
CTX-M-113	*Shigella spp*	AEM44653.1
OXY-1-9	*Klebsiella oxytoca*	ALC79258.1
CTX-M-223	*Escherichia coli*	AXV45344.1
CTX-M-174	*Escherichia coli*	ALM96713.1
CTX-M-67	*Escherichia coli*	ABS90365.1
CTX-M-58	*Escherichia coli*	ABM97538.1
OXY-6-2	*Klebsiella oxytoca*	BAS39381.1
OXY-2-1	*Klebsiella oxytoca*	CAI43414.1
OXY-2-10	*Klebsiella oxytoca*	ACV44456.1
CTX-M-52	*Klebsiella pneumoniae*	ABB17185.1
